# Dietary challenges differentially affect activity and sleep/wake behavior in *mus musculus*: Isolating independent associations with diet/energy balance and body weight

**DOI:** 10.1371/journal.pone.0196743

**Published:** 2018-05-10

**Authors:** Isaac J. Perron, Brendan T. Keenan, Karthikeyani Chellappa, Nicholas F. Lahens, Nicole L. Yohn, Keith R. Shockley, Allan I. Pack, Sigrid C. Veasey

**Affiliations:** 1 Center for Sleep and Circadian Neurobiology, Perelman School of Medicine, University of Pennsylvania, Philadelphia, Pennsylvania, United States of America; 2 Institute for Diabetes, Obesity, and Metabolism, Perelman School of Medicine, University of Pennsylvania, Philadelphia, Pennsylvania, United States of America; 3 Department of Systems Pharmacology and Translational Therapeutics, Perelman School of Medicine, University of Pennsylvania, Philadelphia, Pennsylvania, United States of America; 4 Department of Psychiatry, Perelman School of Medicine, University of Pennsylvania, Philadelphia, Pennsylvania, United States of America; 5 Biostatistics and Computational Biology Branch, National Institute of Environmental Health Sciences, National Institutes of Health, Research Triangle Park, North Carolina, United States of America; State University of Rio de Janeiro, BRAZIL

## Abstract

**Background and aims:**

Associated with numerous metabolic and behavioral abnormalities, obesity is classified by metrics reliant on body weight (such as body mass index). However, overnutrition is the common cause of obesity, and may independently contribute to these obesity-related abnormalities. Here, we use dietary challenges to parse apart the relative influence of diet and/or energy balance from body weight on various metabolic and behavioral outcomes.

**Materials and methods:**

Seventy male mice (*mus musculus)* were subjected to the diet switch feeding paradigm, generating groups with various body weights and energetic imbalances. Spontaneous activity patterns, blood metabolite levels, and unbiased gene expression of the nutrient-sensing ventral hypothalamus (using RNA-sequencing) were measured, and these metrics were compared using standardized multivariate linear regression models.

**Results:**

Spontaneous activity patterns were negatively related to body weight (p<0.0001) but not diet/energy balance (p = 0.63). Both body weight and diet/energy balance predicted circulating glucose and insulin levels, while body weight alone predicted plasma leptin levels. Regarding gene expression within the ventral hypothalamus, only two genes responded to diet/energy balance (*neuropeptide y* [*npy*] and *agouti-related peptide* [*agrp*]), while others were related only to body weight.

**Conclusions:**

Collectively, these results demonstrate that individual components of obesity—specifically obesogenic diets/energy imbalance and elevated body mass—can have independent effects on metabolic and behavioral outcomes. This work highlights the shortcomings of using body mass-based indices to assess metabolic health, and identifies novel associations between blood biomarkers, neural gene expression, and animal behavior following dietary challenges.

## Introduction

Obesity and overweightness are conditions of excessive fat or body weight, typically quantified by body mass index (BMI: weight / height^2^) in humans. Currently, over 70% of American adults are overweight (BMI > 25 kg/m^2^) with roughly half of those people considered obese (BMI > 30 kg/m^2^) [1]. Obesity is a risk factor for numerous morbidities, including hypertension, type II diabetes, neurological disorders (such as depression), and sleep disorders (such as sleep apnea) [2–4]. Therefore, there is great incentive to determine the links between obesity and physiological dysregulation.

Excess body weight *per se* is not the only predictor of molecular, metabolic, and behavioral abnormalities. Some studies have tracked obese individuals shortly after bariatric surgery (<4 weeks), when weight loss has initiated but these people are still considered obese (BMI > 30 kg/m^2^). These patients exhibit reductions in hypertension, normalization of many blood metabolite levels, and significant improvements in subjective sleepiness and alertness prior to significant weight loss [5–7]. Second, moderate diet-induced weight loss in humans improves cardiometabolic health outcomes [8, 9]. Third, studies with diet-induced obese (DIO) mice following weight loss show similar effects to humans. DIO mice switched to a leptogenic diet show normalized glucose tolerance and insulin sensitivity, even though they weigh significantly more than lean controls [10]. Furthermore, our previous work using a diet switch feeding paradigm found that mice gaining weight on high-fat diet exhibit increased total sleep time and sleep/wake fragmentation compared to overweight mice losing weight due to regular chow consumption, despite these two groups of mice having nearly identical body weight at the time of recording [11]. Using linear regression modeling, we demonstrated that body weight and diet/energy balance independently influence sleep/wake architecture [11]. Collectively, these studies demonstrate that diet/energy balance could also influence certain obesity-related physiological abnormalities.

These studies highlight underexplored areas of research, specifically related to obesity-related pathogenesis and its reversibility. First, how are metabolic parameters affected by initial consumption of high-fat diet, and do these parameters normalize in obese animals following weight loss? Second, what is the relative contribution of diet/energy balance vs body mass in mediating these changes? Third, can we build hypothesis-generating models assessing changes in gene expression in relevant brain regions, biomarkers in the blood, and spontaneous sleep/wake and locomotor activity and isolate their relationship to diet/energy balance and body weight? The present study is focused on addressing these broad questions.

Following high-fat diet-induced weight gain, many peripheral organs release hormones (e.g., insulin and leptin), which signal to the central nervous system (CNS) to reduce caloric intake [12, 13]. However, hormone resistance develops within three days of overfeeding, causing an increase in circulating hormone levels but blunted downstream signaling within the CNS [14, 15]. One of the critical brain areas responsible for sensing and responding to these hormones is the ventral hypothalamus (VH), which contains the arcuate nucleus (ARC), ventromedial nucleus (VMN), and paraventricular nucleus (PVN). These heterogeneous populations of neurons adjoin the 3^rd^ ventricle, thus allowing entry of these hormones into the CNS [13, 16, 17]. Furthermore, these VH nuclei are defined both anatomically and by the neuropeptides they express, many of which have roles in energy balance [18]. Importantly, many VH nuclei, including the ARC, send projections to both intra- and extra-hypothalamic brain areas important for regulating behavior, including locomotor activity and sleep and wakefulness [19, 20]. Furthermore, VH neurons express functional hormone receptors, including for insulin and leptin, and their activation alters the electrochemical properties of these neurons, thereby affecting the synthesis and release of neuropeptides [17, 18, 21]. Taken together, the VH contains a heterogeneous population of neurons and neuropeptides that work in concert to regulate complex behaviors such as food intake, energy expenditure, and sleep/wake. However, it is unclear how the input signals to the VH, the VH itself, and downstream behaviors are relatively influenced by changes in diet/energy balance and body weight.

We hypothesize that identifying unique associations of blood hormones, VH gene expression, and behavior to diet/energy balance and body mass to will reveal novel relationships between these physiological parameters. We found that sleep/wake behavior, but not spontaneous activity patterns, were affected by diet/energy balance; these behaviors are highly correlated under normal conditions [11, 22]. Furthermore, we use linear modeling approaches to relate circulating metabolites, neural gene expression, and behavior, finding some VH genes and blood biomarkers correlate with spontaneous locomotor activity while others correlate with electroencephalography (EEG)-measured sleep/wake behavior.

## Methods

### Animals

Male C57BL/6J mice (12–14 weeks of age) were purchased directly from Jackson Laboratories (#664; Bar Harbor, ME). The Institutional Animal Care and Use Committee (IACUC) at the University of Pennsylvania approved all experimental procedures prior to initiation of these studies. Mice were housed at 23°C on a 12:12 h light:dark cycle, with lights-on at 7:00am. Light intensity was 100–150 lux during lights ON (Zeitgeber [ZT] 0–12), and 0 lux during lights OFF (ZT 12–24).

### Diet Switch protocol

Upon arrival to our facility, mice were weighed (Week 0), ear tagged, and group-housed while consuming either regular chow (RC; 5001, Lab Diet, St. Louis, MO) or high-fat diet (HFD; D12451, Research Diets, New Brunswick, NJ). RC is composed predominately of corn, soybean meal, and vitamin supplements and contains 13.5% calories from fat and 3.35 kcal/g, while the HFD (predominately composed of soybean oil and lard) contains 45% calories from fat and 4.73 kcal/g. We chose these diets because of their well-studied effects on metabolic and behavioral changes in C57BL/6 mice (including our previous work) [11]. After 7–9 weeks, mice were single housed (one week acclimation) then placed into homecage locomotor boxes to quantify spontaneous activity (3 days continuous recording). We then randomized mice to either consume the alternate diet (Diet Switch [DS]; RC → HFD or HFD → RC groups) or maintenance on current diet (No Diet Switch [NoDS]; RC NoDS or HFD NoDS groups). Food intake and body weight were measured daily between ZT2-3. On the 7^th^ day Post-DS, mice were kept awake from ZT3-6 using novel objects to control for behavioral state differences that may result from excessive sleepiness in HFD-fed mice compared to RC-fed mice [11, 23]. All animals were sacrificed between ZT6-7 by cervical dislocation and decapitation. Food and water were provided *ad libitum* at all times, including during the forced wakefulness prior to sacrifice.

All data is generated from two cohorts of animals. The first cohort (80 animals) represents a novel data set which is the primary focus of this manuscript. Nine mice never completed the protocol (due to fight wounds or dehydration by malfunctioning sipper tubes) and one mouse lost ~10% body mass in the ‘positive energy balance’ group (RC → HFD); therefore, 70 mice were used in total. The group sizes were: RC NoDS, n = 16; RC → HFD, n = 21; HFD → RC, n = 19; HFD NoDS, n = 14. The second cohort of animals was from a previously published manuscript observing EEG-recorded sleep behavior following the DS feeding paradigm; the experimental methods are similar and are detailed in [11].

### Homecage locomotor activity and sleep/wake estimation

Spontaneous activity patterns were recorded using infrared beam splitting locomotor boxes (Omnitech Electronics, Columbus, OH). We recorded the number of beams broken in both x- and y-planes, representing the total activity of the animal; Z-axis measurements were not measured. Total activity counts were exported into 10 second bins, which was used to quantify activity and estimate sleep/wake architecture as previously described [22]. Briefly, 40-seconds or longer of inactivity (0 beams broken) is defined as ‘sleep’ while everything else is defined as ‘wake’; this algorithm has >90% agreement with EEG-recorded sleep in healthy, lean mice [22]. These calculations were performed in MATLAB.

### Blood and brain collection

Immediately after sacrifice, blood was transferred to heparin-containing microtainers (365965, Fisher Scientific, Waltham, MA) and spun at room temperature for 10 min at 10,000 rpm. The supernatant was pipetted to clean Eppendorf tubes and stored at -80°C. Brains were immediately dissected and flash frozen in dry ice-cold isopentane (2-mehtylbutane, Fisher Scientific). Whole brains were stored at -80°C until further use.

### Ventral hypothalamus (VH) mRNA purification

Whole brains were mounted and sectioned using a cryostat. Beginning at -1.3 mm anteroposterior of bregma, three 400 μm sections were transferred to a cold RNAse-free microscope slide. One punch (1.2 mm diameter) of the ventral hypothalamus (VH; enriched with ARC, VMN, and PVN nuclei, but not the lateral hypothalamus [LH]) per section was transferred to an RNAse-free 2.0 mL Eppendorf tube. The RNeasy mini prep (74104, Qiagen, Frederick, MA) was used to isolate mRNA and the eluted mRNA product was DNAse treated (2224G1, AM1931, Fisher Scientific) per the manufacturers’ protocol. Concentration of purified mRNA was measured using the Nanodop v1.0 (Fisher Scientific). To ensure accuracy of punches, we measured gene expression of two ARC genes (*agouti-related peptide [agrp]* and proopiomelanocortin [*pomc]*) and one LH gene (hypocretin/orexin, or *hcrt*). Punches that did not have 5-fold enrichment of average *agrp* and *pomc* compared to *hcrt* were omitted from RNA-seq and future RT-qPCR validation studies. Of 70 brains sectioned, 8 punches did not meet these quality standards.

### RNA-sequencing (RNA-seq)

To reduce technical and biological variability, two mRNA samples per condition were randomly chosen and pooled equally (300 ng mRNA total). A total of 24 pools (n = 6 per condition) were ligated with unique sequencing adapters using the TruSeq Stranded mRNA kit (RS-122-2101, Illumnia, San Diego, CA) following the manufacturer’s protocol for polyA enrichment. Each sample was quality checked for concentration, molarity, and length distribution using the Qubit (3.0, ThermoFisher), BioAnalyzer (2100, Agilent, Santa Clara, CA), and Kapa (KK4873, Kapa Biosystems, Wilmington, MA), respectively. Three samples did not meet quality control standards (one each from RC NoDS, HFD → RC, and HFD NoDS conditions) and were not loaded onto the sequencer. The remaining 21 samples were equimolarly pooled and run thrice on the HiSeq 4000 system (Illumina, ~350 million reads per run, 100 bp read, single end). To merge fastq and BAM files from the three runs, we used Picard Tools (v2.7.1, http://broadinstitute.github.io/picard/). Alignment of fastq files to the mouse reference genome (mm9 build) was conducted with STAR (v2.5.1b) using gene models from Ensembl v67 genome annotation [24, 25]. Over 95% of reads were aligned to the genome for each sample. To normalize the data, we used the Pipeline of RNA-Seq Transformations, or PORT (v0.8.2a-beta, https://github.com/itmat/Normalization) [26]. Briefly, PORT recognizes potential confounding factors (e.g., ribosomal RNA and mitochondrial DNA) and omits these reads from the normalization process. Importantly, our samples had normal ranges and distributions of both ribosomal RNA (2.28–6.05%) and mitochondrial DNA (0.5–1.8%; see S1 Table). Next, PORT identifies the input sample with the fewest number of gene-mapping reads and randomly re-samples each of the other datasets down to this minimum read count. This re-sampling approach accounts for batch effects and differences in sequencing depth between the samples, and allows for direct comparison between samples [26]. All samples were re-sampled to 20.98 million reads; the original number of reads per sample is shown in S1 Table. All data generated and analyzed are available in the Gene Expression Omnibus repository (GSE104709; https://www.ncbi.nlm.nih.gov/geo/query/acc.cgi?&acc=GSE104709).

There were 21,517 unique genes detected, although many of these genes had very low expression levels. Therefore, we implemented two filtering approaches to remove the least expressed genes [27, 28]. The ‘low filter’ removed all genes averaging <5 reads per sample; 15,833 genes remained, accounting for 99.96% of total gene expression. The ‘high filter’ removed all genes averaging <50 reads per sample; 12,752 genes remained, accounting for 99.59% of total gene expression. The unfiltered and filtered data sets were then analyzed using two distinct statistical approaches to discover differentially expressed genes. First, the LimmaVoom package (v3.5, www.bioconductor.org/packages/limma) is a linear regression modeling approach originally designed for microarrays and now adapted for RNA-seq. Second, we used the microarray analysis of variance, or MAANOVA package (v3.5, www.bioconductor.org/packages/release/bioc/html/maanova.html), which was also originally designed for microarray analysis. For MAANOVA, we first log2-transformed expression values to approximate normally distributed data and then used permutation analysis (1000 total) to calculate p-values. Three distinct comparisons were tested: 1) RC NoDS v HFD NoDS, to discover genes affected by obesity; 2) RC → HFD v HFD → RC, to discover genes affected by diet/energy balance; and 3) all four groups simultaneously (only possible with MAANOVA). Differentially expressed genes (DEG) were considered significant at a false discovery rate (FDR, or q-value) < 0.15. Key findings were confirmed by RT-qPCR.

### Gene set enrichment analysis (GSEA)

We used gene set enrichment analysis (GSEA, v3.0, http://www.broad.mit.edu/gsea) to discover differences in enriched pathways between DS conditions [29]. This approach considers the entire matrix of gene expression data, ranks the genes based off the magnitude of differential expression, and identifies coherent expression patterns against pre-defined lists for established biochemical pathways. We used the ‘high filter’ data set as input (see previous section). GSEA annotations are based on human gene data sets, so all mouse gene symbols were converted to human gene symbols using the Mouse Genome Informatics database from the JAX labs website (HOM_MouseHumanSequence.rpt); 1495 genes did not have human homologs and were omitted from GSEA. Therefore, gene expression data matrix for 11,257 genes were input into GSEA and tested against the Hallmark Gene Set (v6.0, MSigDB) using ‘gene_set’ permutations (1000 total). We tested both raw and log2-transformed data, and used a family-wise error rate (FWER) cut-off of p<0.05. Both raw and log2-transformed data returned the same top six significantly enriched pathways (S2 Table).

### RT-qPCR

Purified mRNA was converted to cDNA using the High-Capacity RNA-to-cDNA kit (4387406, ThermoFisher). RT-qPCR was performed with pre-mixed Taqman primer/probes (4331182, ThermoFisher; see S3 Table) and Taqman master mix (4369016, ThermoFisher) using 4 ng cDNA per well (10 μL reactions). 384-well plates were robotically loaded using the Biomek 3000 (Beckman Coulter, Indianapolis, IN) and analyzed using the 7900HT Fast Real-Time PCR System (Fisher Scientific). Each gene of interest was normalized to the geometric mean of three housekeeping genes (*18s*, *rplp0*, and *actb*); the Ct values of these housekeeping genes did not differ between dietary conditions. Statistical tests and linear modeling were conducted using ΔΔCt values to omit floor effects (normalized to RC NoDS) and data are presented as percent change from RC NoDS [30].

### Blood biomarkers

Plasma was thawed on ice, with no more than 2 freeze-thaw cycles prior to all measurements. Glucose was measured using the OneTouch Ultra Mini (LifeScan, Wayne, PA). Leptin and insulin were measured using the Luminex multiplex kit (MMHMAG-44K-03, EMD Millipore). The thyroid hormones T3 and T4 were also measured using the Luminex multiplex kit (RTHYMAG-30K-02, EMD Millipore), and TSH was quantified with ELISA (EKU07683, Biomatik, Wilmington, DE).

### Statistical methods

All statistical analyses and figures were conducted in PRISM (GraphPad, La Jolla, CA), except linear regression, LimmaVoom, and MAANOVA analyses were conducted in R (v3.3.3, www.R-project.org). To compare individual group differences across the four dietary conditions, we used a one-way analysis of variance (ANOVA) with Tukey’s post-hoc correction. When data were stratified across multiple time points (e.g., spontaneous activity), we used a repeated-measure two-way ANOVA with ‘time’ as the within-subjects variable and ‘dietary condition’ as the between-subjects variable. In order to isolate ‘body weight’ from ‘diet and/or energy balance’ effects on the outcome metrics, we used two linear modeling approaches. First, we input data only from the weight-matched DS conditions (RC → HFD and HFD → RC) with absolute body weight (in grams) and an arbitrary categorical variable indicating condition (RC → HFD = 0 and HFD → RC = 1) as the only two predictors. Second, we used acute weight changes (Δbody weight; percent weight change from Pre-DS to Post-DS) as an estimate for diet/energy balance because positive energy balance (caloric consumption > energy expenditure) causes weight gain while negative energy balance (caloric consumption < energy expenditure) causes weight loss [31]. Therefore, we used acute diet-induced weight changes as a proxy for energy balance; the limitations of this assumption are discussed later. Along with absolute body weight (Day 7 Post-DS, in grams) as a predictor, we made joint linear regression models containing data from all animals. Primary results from linear regression models are presented as estimated beta-coefficients (β) and standard errors, representing the expected change in outcome for a 1 unit increase in predictor (e.g., 1 gram for body weight, or 1% for Δbody weight). In addition, to provide comparable estimates between predictors, we calculated standardized β estimates (Stdβ_xy_) equal to the expected Z-score change in outcome for a 1 standard deviation increase in the predictor. Statistical significance in these analyses was based on the β-coefficient of interest, testing the null hypothesis of no association (H_0_: β = 0 vs. H_A_: β ≠ 0). Significance was achieved at p<0.05, although trends towards significance and actual p-values were reported where appropriate. Detailed statistical results are shown in S4 Table. All data used for analysis is publically available on Dryad (doi:10.5061/dryad.qm01v75).

## Results

### Diet Switch (DS) generates mice of similar body weight but opposing energetic status

**Fig 1:**
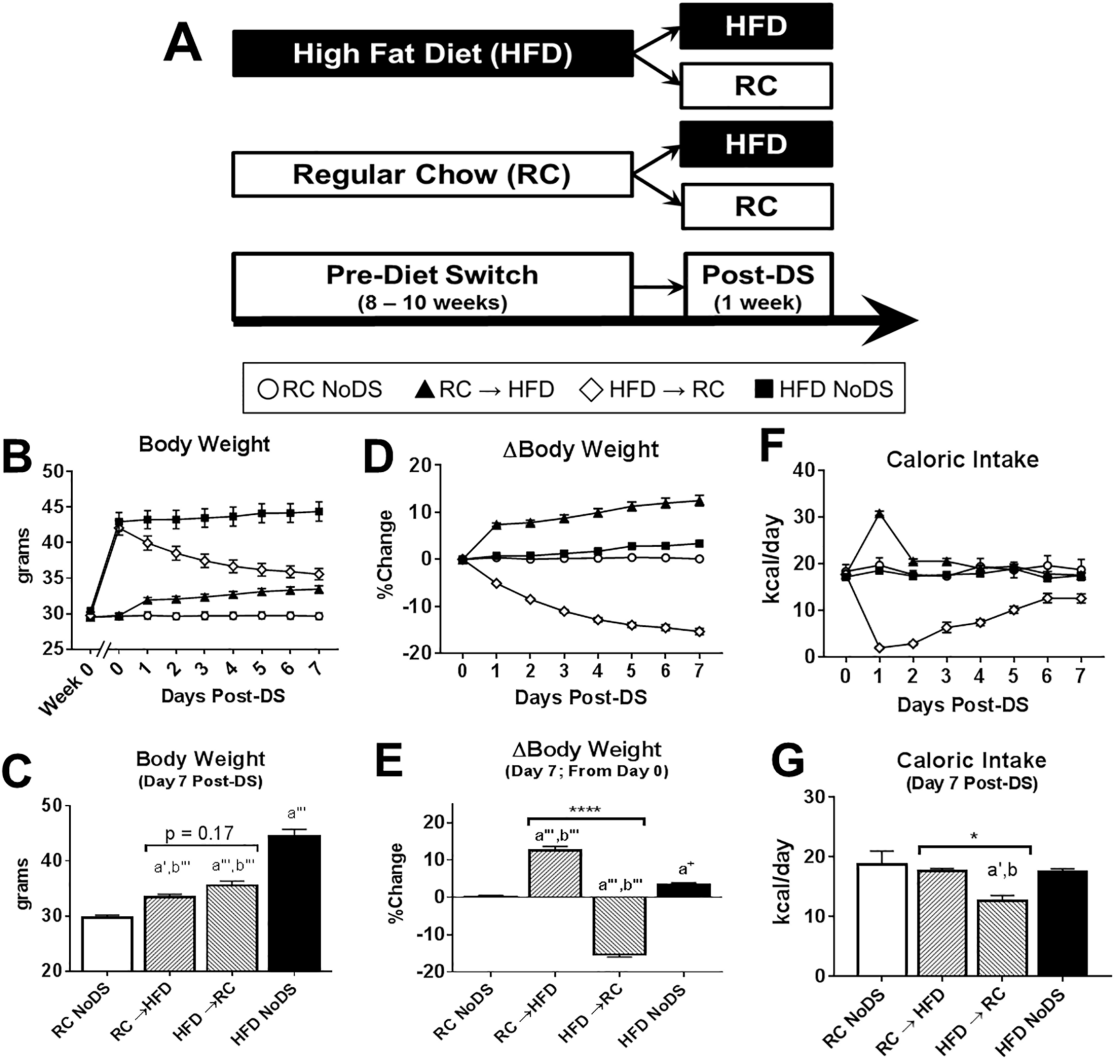
Diet Switch (DS) feeding paradigm isolates ‘body weight’ from ‘diet and/or energy balance’ factors. (A) Experimental design schematic. Mice consume either RC or HFD for 8–10 weeks (Pre-DS), then are randomized to either continue consuming their respective diet (RC NoDS and HFD NoDS) or switched to the alternate diet (RC → HFD and HFD → RC). All mice were sacrificed Day 7 Post-DS. (B) Absolute body weight across the study. (C) Absolute body weight at Day 7 Post-DS. (D) Percent change in body mass (ΔBody Weight) normalized to Pre-DS weights (Day 0 Post-DS). (E) ΔBody weight on Day 7 Post-DS (normalized to Pre-DS weight). (F) Caloric intake from Pre-DS (Day 0) to Day 7 Post-DS. (G) Average caloric intake on Day 7 Post-DS. *p<0.05, ****p<0.0001 comparing RC → HFD vs HFD → RC; a^+^: p<0.1, a: p<0.05, a’: p<0.01, a”: p<0.001, a”‘: p<0.0001 compared to RC NoDS; b^+^: p<0.1, b: p<0.05, b’: p<0.01, b”: p<0.001, b”‘: p<0.0001 compared to HFD NoDS. Sample sizes for B-E: [RC NoDS, n = 16; RC → HFD, n = 21; HFD → RC, n = 19; HFD NoDS, n = 14]. Sample sizes for F-G: [RC NoDS, n = 15; RC → HFD, n = 21; HFD → RC, n = 18; HFD NoDS, n = 14].

HFD consumption stimulates weight gain (positive energy balance) and eventually leads to obesity, making it difficult to parse apart diet, energy balance, and body mass effects on metabolic and behavioral metrics. Here, we use the DS feeding paradigm to isolate body mass effects from diet and/or energy balance (Fig 1A). Briefly, all mice are fed either RC or HFD for 8–10 weeks, then subsets of mice are randomized to consume the opposite diet (RC → HFD and HFD → RC) while other diet maintenance groups (RC ‘No Diet Switch’ [NoDS] and HFD NoDS) are used as reference (Fig 1B and 1C). During the one week Post-DS, lean mice switched to HFD (RC → HFD group) gain 12.5% body mass on average, while HFD-fed obese mice switched to RC (HFD → RC group) lose 15.3% body mass (Fig 1D and 1E). At the study’s conclusion (Day 7 Post-DS), the two DS groups have statistically similar weight (Fig 1C: p = 0.17) while each DS group weighs more than RC NoDS and less than HFD NoDS controls. Therefore, we can use linear models to compare these two DS groups to isolate body weight effects from diet and/or energy balance effects on the reported metrics.

### Caloric intake

RC and HFD drives weight changes, in part, by differences in caloric intake. Therefore, we monitored daily caloric intake before the DS (Day 0) and every day Post-DS (Fig 1F). We found that caloric intake was similar between RC NoDS and HFD NoDS groups at all time points. RC → HFD animals increased caloric consumption the first day Post-DS, likely due to a combination of increased palatability and greater caloric density (e.g., greater number of calories per gram consumed) of HFD compared to RC. However, energy intake returned to RC NoDS and HFD NoDS levels by the seventh day (Fig 1F and 1G: p = 0.90 and p>0.99, respectively). Conversely, HFD → RC animals drastically decreased intake the first day which remained lower than all other groups by final day (Fig 1G: p<0.01 compared to RC NoDS, p<0.05 compared to HFD NoDS, p<0.05 compared to RC → HFD). Linear modeling of energy intake revealed a significant relationship to diet/energy balance (p<0.0001) but not body mass (p = 0.35) when comparing the two DS groups. Therefore, diet/energy balance effects may be mediated in part by caloric intake, but cannot explain associations with body mass.

### Locomotor activity

**Fig 2:**
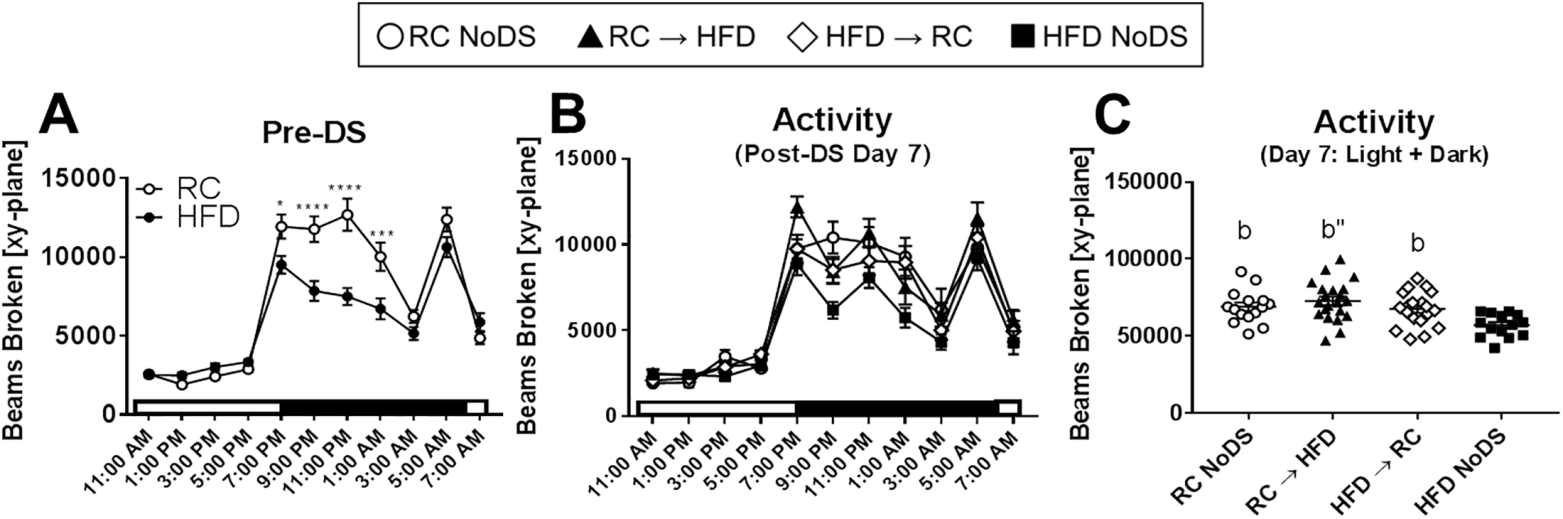
Spontaneous locomotor activity is only reduced in HFD NoDS compared to all other conditions. (A) Pre-DS locomotor activity shows HFD-fed mice are less active compared to RC-fed controls. (B) Locomotor activity counts in 2-hour time bins on Day 7 Post-DS. (C) Aggregate (24-hour) locomotor activity counts Day 7 Post-DS. All conditions are significantly more active than HFD NoDS. Note that body weight and food intake were measured daily between 9–10 am (ZT2-3), so locomotor data are omitted during the ZT2-4 time window. *p<0.05, ***p<0.001, ****p<0.0001 comparing RC v HFD at indicated time point; b: p<0.05, b”: p<0.001 compared to HFD NoDS. RC NoDS, n = 15; RC → HFD, n = 21; HFD → RC, n = 19; HFD NoDS, n = 14.

Our previous work found that RC → HFD mice exhibit profound hypersomnolence compared to HFD → RC mice, even though these two groups had similar body weight at the time of the recording [11]. Since locomotor activity and total sleep/wake amount are highly correlated [22], we tested how spontaneous locomotor patterns would be affected by the DS feeding paradigm. Prior to the DS, HFD-fed mice were significantly less active during the beginning of the dark phase (Fig 2A), which coincides with the hypersomnolence phenotype observed in DIO mice [11]. Next, we estimated total wakefulness from the locomotor data (using the ‘40-second’ cut-off threshold; see Methods), but found that these robust differences disappeared (Panel C in S1 Fig). We also tried different inactivity cut-off thresholds (20-seconds, 30-seconds, and 50-seconds) to estimate wakefulness, but none of these estimates agreed with EEG-measured data (S1 Fig). Therefore, we did not use locomotor-estimated sleep/wake metrics for any further analyses.

One week Post-DS, RC NoDS mice were still significantly more active than HFD NoDS mice (Fig 2B and 2C: p<0.05 for both). Furthermore, HFD → RC animals exhibited statistically greater locomotor activity compared to HFD NoDS mice (Fig 2C: p<0.05), indicating that one week of RC is sufficient to normalize locomotor patterns. Surprisingly, RC → HFD animals, which exhibit similar wake time compared to HFD NoDS mice [11], had significantly greater spontaneous activity compared to HFD NoDS mice (Fig 2C: p<0.001), indicating a decoupling of locomotor and sleep/wake patterns. Indeed, activity was not related to either body mass or diet/energy balance (Table 1), whereas wake time was associated with both of these metrics [11]. Therefore, two highly correlated behaviors—spontaneous activity and sleep/wake patterns—show dissimilar associations with body mass and diet/energy balance as tested by the DS feeding paradigm.

**Table 1:** Linear modeling results comparing weight-matched DS conditions.

Metric	Linear Regression Models Only RC → HFD and HFD → RC groups					
	ANOVA		Body Weight		Diet/Energy Balance	
	*F-value*	*p-value*	*β* *(SEM)*	*p-value*	*β* *(SEM)*	*p-value*
***Animal Behavior***						
**Caloric Intake** 24 Hour	**12.8**	**<0.0001**	-0.187 (0.199)	0.35	**4.52** **(1.01)**	**<0.0001**
**Activity** Dark	2.33	0.11	-901.7 (573.9)	0.12	2908.9 (3284.3)	0.38
**Activity** Light	0.67	0.52	-252.6 (243.6)	0.31	172.8 (1394.0)	0.90
**Activity** 24 Hour	2.35	0.11	-1154.3 (695.2)	0.11	3081.6 (3978.3)	0.44
***VH Gene Expression***						
***Agrp***	**12.4**	**0.0002**	**0.128** **(0.040)**	**0.0033**	**1.163** **(0.244)**	**<0.0001**
***Npy***	**25.1**	**<0.0001**	**0.108** **(0.028)**	**0.0007**	**1.214** **(0.174)**	**<0.0001**
***Cartpt***	0.36	0.70	-0.027 (0.032)	0.41	-0.072 (0.199)	0.72
***Pomc***	1.88	0.17	0.066 (0.054)	0.24	-0.300 (0.335)	0.38
***Trh***	0.49	0.62	-0.004 (0.045)	0.93	0.237 (0.275)	0.40
***Sbno2***	0.69	0.51	0.0011 (0.252)	0.96	0.170 (0.156)	0.28
***Serpina3n***	0.25	0.78	-0.0019 (0.0319)	0.95	0.121 (0.197)	0.54
***Blood Biomarkers***						
**Glucose**	**29.6**	**<0.0001**	**4.71** **(1.90)**	**0.018**	**83.78** **(10.89)**	**<0.0001**
**Insulin**	**4.88**	**0.016**	187.8 (113.6)	0.11	**2045.2** **(665.2)**	**0.0049**
**Leptin**	1.6	0.22	216.7 (149.2)	0.16	1259.2 (869.9)	0.16
**T4**	0.83	0.45	-6939 (5395)	0.21	-1.2x10^4^ (3.1x10^4^)	0.71

Estimates are from joint regression models containing both weight factors. ANOVA: Indicates overall significance of linear model. *β*: β-estimate and standard error indicates expected change in outcome for a 1 unit increase in predictor. SEM: Standard Error of the Mean. Highlighted cells indicate significance at p<0.05. Note that VH gene expression values are modeled using RT-qPCR results from sequenced tissue of individual animals to maximize sample size. *cartpt*: cocaine- and amphetamine regulated transcript; *serpina3n*: serine (or cysteine) peptidase inhibitor clade A member 3N; *sbno2*: strawberry notch homolog 2; *trh*: thyrotropin-releasing hormone; *agrp*: agouti-related peptide; *npy*: neuropeptide y; *pomc*: proopiomelanocortin; T4: Thyroxine.

### Ventral hypothalamus (VH) gene expression

#### RNA-sequencing results

The VH responds to circulating cues to affect energy expenditure, locomotor activity, and sleep/wake behavior [19, 20]. Therefore, we used RNA-seq to perform unbiased quantification of all protein-coding transcripts within the VH. After acquiring locomotor activity, mice were sacrificed between ZT6-7 and coding mRNA from the VH was purified and sequenced (see Methods). There were 21,517 unique genes aligned, but low-expressed transcripts were removed using two data filtering approaches: 1) a ‘Low Filter’, removing all genes averaging <5 reads per sample (15,833 remaining genes), and 2) a ‘High Filter’, removing all genes averaging <50 reads per sample (12,752 remaining genes). We then tested for DEG using two statistical packages: MAANOVA and LimmaVoom (Table 2). MAANOVA results varied depending on the data input, reporting significance for some low-expressed genes (e.g., *tk1* and *cldn2*) and increasing the number of DEG as the number of multiple comparisons decreased. Conversely, LimmaVoom results were extremely consistent for all three data inputs, reporting the same six genes as significantly different between RC NoDS v HFD NoDS comparisons (Table 2). Interestingly, both MAANOVA and LimmaVoom found the same six DEGs using the ‘High Filter’ data input. Therefore, we focused on these six genes (*trh*, *npy*, *cartpt*, *agrp*, *serpina3n*, and *sbno2*) for follow-up analyses.

**Table 2:** Differentially expressed genes (DEG) from RNA-sequencing analysis.

Statistical Approach	Data Filtering						Common
	No Filter		Low Filter		High Filter		
**MAANOVA**	*trh* *npy* *tk1* *cldn2* *atxn7l2*	q_*¥*_ = 0.024 q_*ϕ*_ = 0.094 q_*¥*_ = 0.075 q_*ϕ*_ = 0.10 q_*§*_ = 0.14 q_*§*_ = 0.14 q_*ϕ*_ = 0.12	*trh* *npy* *cartpt* *sbno2* *tk1* *cldn2* *ikzf3* *atxn7l2* *rbm3*	q_*¥*_ = 0.005 q_*ϕ*_ = 0.023 q_*¥*_ = 0.036 q_*ϕ*_ = 0.032 q_*¥*_ = 0.133 q_*¥*_ = 0.133 q_*§*_ = 0.049 q_*ϕ*_ = 0.12 q_*§*_ = 0.049 q_*ϕ*_ = 0.12 q_*¥*_ = 0.075 q_*ϕ*_ = 0.059 q_*ϕ*_ = 0.059 q_*ϕ*_ = 0.12	*trh* *npy* *cartpt* *sbno2* *serpina3n* *c4b* *atxn7l2* *rbm3* *agrp*	q_*¥*_ < 1x10^-6^ q_*ϕ*_ = 0.002 q_*¥*_ = 0.006 q_*ϕ*_ = 0.004 q_*¥*_ = 0.059 q_*ϕ*_ = 0.12 q_*¥*_ = 0.059 q_*ϕ*_ = 0.12 q_*¥*_ = 0.083 q_*¥*_ = 0.10 q_*ϕ*_ = 0.12 q_*ϕ*_ = 0.030 q_*ϕ*_ = 0.079 q_*ϕ*_ = 0.12	*trh* *npy* *atxn7l2*
**Limma Voom**	*trh* *npy* *cartpt* *serpina3n* *agrp* *sbno2*	q_*¥*_ = 1.5x10^-5^ q_*¥*_ = 6.4x10^-5^ q_*¥*_ = 0.002 q_*¥*_ = 0.004 q_*¥*_ = 0.007 q_*¥*_ = 0.12	*trh* *npy* *cartpt* *serpina3n* *agrp* *sbno2*	q_*¥*_ = 1.9x10^-5^ q_*¥*_ = 7.7x10^-5^ q_*¥*_ = 0.002 q_*¥*_ = 0.004 q_*¥*_ = 0.008 q_*¥*_ = 0.12	*trh* *npy* *cartpt* *serpina3n* *agrp* *sbno2*	q_*¥*_ = 2.1x10^-5^ q_*¥*_ = 8.7x10^-5^ q_*¥*_ = 0.002 q_*¥*_ = 0.004 q_*¥*_ = 0.008 q_*¥*_ = 0.11	*trh* *npy* *cartpt* *serpina3n* *agrp* *sbno2*
***Common***	*trh* *npy*		*trh* *npy* *cartpt* *sbno2*		*trh* *npy* *cartpt* *serpina3n* *agrp* *sbno2*		***trh*** ***npy***

Genes with false discovery rate (q-value) < 0.15 for the following comparisons: RC NoDS v HFD NoDS (designated by ¥); RC → HFD and HFD → RC groups (§); or, all four groups (Φ; MAANOVA only). This analysis reveals 6 candidate genes: *cartpt*: cocaine- and amphetamine regulated transcript; *serpina3n*: serine (or cysteine) peptidase inhibitor clade A member 3N; *sbno2*: strawberry notch homolog 2; *trh*: thyrotropin-releasing hormone; *agrp*: agouti-related peptide; *npy*: neuropeptide y.

#### RT-qPCR validation of RNA-seq results

**Fig 3:**
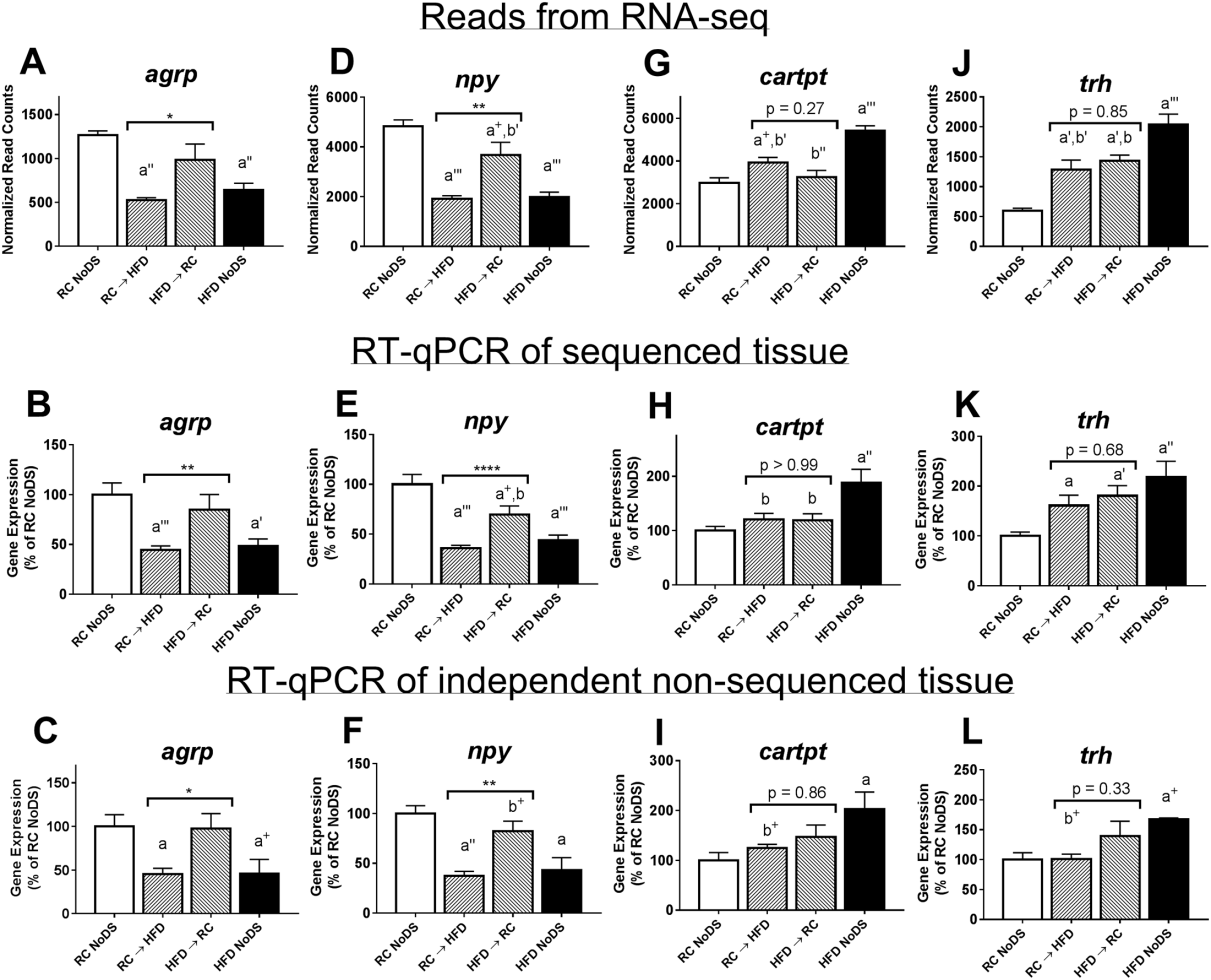
RNA-seq results and RT-qPCR confirmation of VH-expressed genes is consistent between sequenced and non-sequenced tissue. (Top row) Normalized read counts from RNA-seq analysis. (Middle row) RT-qPCR validation using tissue used for RNA-seq. (Bottom row) RT-qPCR validation using independent tissue samples not used for RNA-seq. (A-C) *Agrp* and (D-F) *npy* gene expression is lowest in RC → HFD and HFD NoDS compared to HFD → RC and RC NoDS conditions. (G-I) *Cartpt* and (J-L) *trh* gene expression is highest in HFD NoDS, lowest in RC NoDS, and low to intermediate in both DS conditions. *agrp*: agouti-related peptide; *npy*: neuropeptide y; *cartpt*: cocaine- and amphetamine regulated transcript; *trh*: thyrotropin-releasing hormone. *p<0.05, **p<0.01, ****p<0.0001 comparing RC → HFD vs HFD → RC; a^+^: p<0.1, a: p<0.05, a’: p<0.01, a”: p<0.001, a”‘: p<0.0001 compared to RC NoDS; b^+^: p<0.1, b: p<0.05, b’: p<0.01, b”: p<0.001, b”‘: p<0.0001 compared to HFD NoDS. Sample sizes for top row: [RC NoDS, n = 5; RC → HFD, n = 6; HFD → RC, n = 5; HFD NoDS, n = 5]. Sample sizes for middle row: [RC NoDS: n = 13; RC → HFD: n = 18; HFD → RC: n = 11; HFD NoDS: n = 7]. Samples sizes for bottom row: [RC NoDS: n = 5; RC → HFD: n = 8; HFD → RC: n = 6; HFD NoDS: n = 3].

We validated RNA-seq results using RT-qPCR both from VH tissue used for RNA-seq and from VH tissue from other animals which was not sequenced (Fig 3 and S2 Fig). Overall, we found similar trends with RT-qPCR compared to the RNA-seq. Specifically, *agrp* and *npy* were decreased in both RC → HFD and HFD NoDS groups compared to both RC NoDS and HFD → RC groups (Fig 3A–3F) and linear models showed that *agrp* and *npy* are significantly associated with both body weight and diet/energy balance (Table 1). The other four genes—*trh*, *cartpt*, *serpina3n*, and *sbno2*—showed highest expression in HFD NoDS, lowest expression in RC NoDS, and low to intermediate expression in both DS groups (Fig 3G–3L and Panels A-F in S2 Fig). Since gene expression levels were similar between DS groups (p>0.05 for all), linear models could not be fit for *trh*, *cartpt*, *serpina3n*, and *sbno2* genes (Table 1: p>0.05 for main effect). Last, we quantified expression of two genes for which we did not expect expression differences, specifically proopiomelanocortin (*pomc*) and hypocretin/orexin (h*crt*). Co-expressed with *cartpt* in the ARC, *pomc* does not exhibit altered expression patterns with prolonged HFD [32, 33], while the VH punches intentionally omitted the *hcrt*-expressing neurons in the LH. Indeed, both genes were significantly similar across all conditions (Panels G-L in S2 Fig), and did we not detect any significant main effects with linear modeling (Table 1). Altogether, RNA-seq discovered a short list of DEGs that were validated in a separate experimental cohort. Of the genes that met the ‘filtering’ expression threshold, only two (*agrp* and *npy*) were differentially expressed between weight-matched DS conditions.

#### GSEA to compare DS conditions

Since our analyses only found a short list of DEGs, we used GSEA to discover differentially regulated biochemical signaling pathways because this approach includes expression data from all genes (whether or not they were DEGs). We input RNA-seq gene expression data only from RC → HFD and HFD → RC groups and tested for differences against 50 gene sets representing common biochemical pathways. Of these 50 gene sets, six pathways were determined significant at FWER < 0.05 (S2 Table). ‘Oxidative Phosphorylation’ showed the strongest significant difference, with enrichment in HFD → RC compared to RC → HFD (S2 Table: p<0.0001).

### Blood biomarkers

#### Glucose and insulin

**Fig 4:**
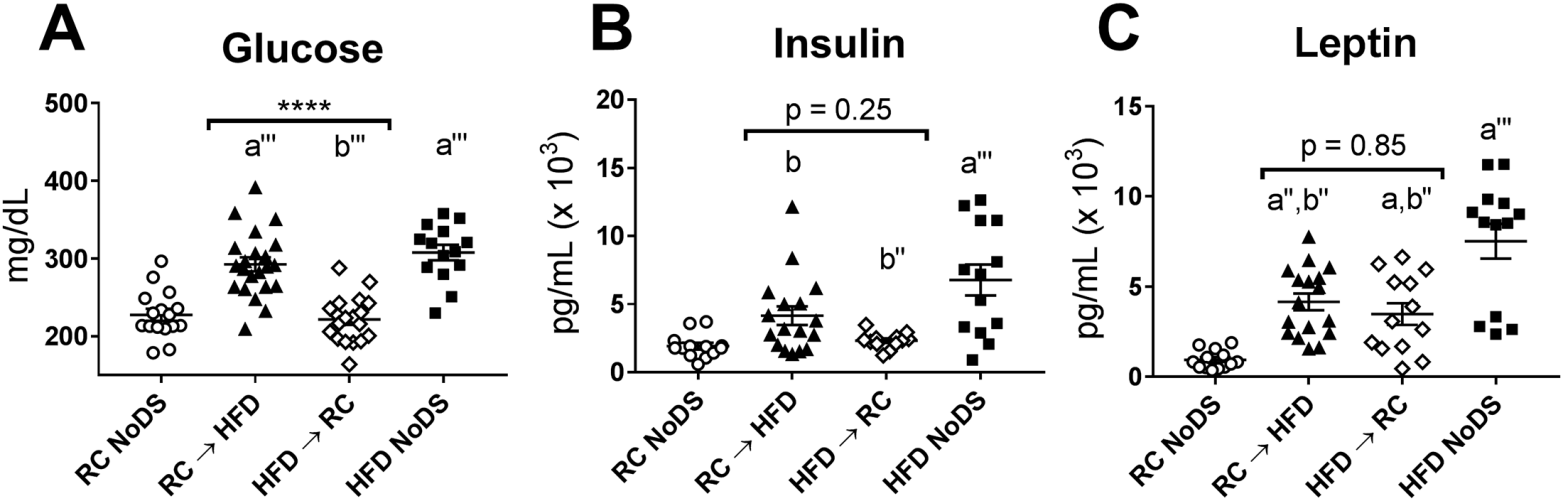
Glucose, insulin, and leptin levels from trunk blood plasma (Day 7 Post-DS). (A) Glucose, (B) insulin, and (C) leptin levels measured from blood plasma, which was collected immediately after sacrifice (ZT6-7). ****p<0.0001 comparing RC → HFD vs HFD → RC; a: p<0.05, a’: p<0.01, a”: p<0.001, a”‘: p<0.0001 compared to RC NoDS; b: p<0.05, b’: p<0.01, b”: p<0.001, b”‘: p<0.0001 compared to HFD NoDS. Sample sizes for glucose: [RC NoDS: n = 16; RC → HFD: n = 21; HFD → RC: n = 19; HFD NoDS: n = 14]. Sample sizes for insulin: [RC NoDS: n = 13; RC → HFD: n = 17; HFD → RC: n = 12; HFD NoDS: n = 13]. Sample sizes for leptin: [RC NoDS: n = 13; RC → HFD: n = 17; HFD → RC: n = 13; HFD NoDS: n = 13].

Obesity is the greatest risk factor for type II diabetes, which is characterized by hyperglycemia and hyperinsulinemia [4]. Therefore, we wanted to test how body mass and diet/energy balance each affect glucose and insulin levels. Glucose levels were significantly elevated in both HFD-consuming groups (RC → HFD and HFD NoDS) compared to both RC-consuming groups (HFD → RC and RC NoDS; Fig 4A). Furthermore, glucose was independently associated with both body weight (Table 1: p = 0.018) and diet/energy balance (p<0.0001). Compared to basal insulin levels (RC NoDS mice), insulin was highest in HFD NoDS mice, intermediate in RC → HFD mice, and unchanged in HFD → RC mice (Fig 4B). Although some RC → HFD animals were hyperinsulinemic compared to HFD → RC animals, these weight-matched DS conditions had statistically similar insulin levels following post-hoc correction (Fig 4B: p = 0.25). Importantly, linear modeling revealed that insulin was strongly associated with diet/energy balance (Table 1: p = 0.0049) but did not show a strong association with body mass (p = 0.11).

#### Leptin

Leptin is synthesized and released from adipocytes, and circulating levels are strongly correlated with body weight [34, 35]. Indeed, we found RC NoDS had the lowest, HFD NoDS had the highest, and both DS groups had intermediate, statistically similar leptin levels (Fig 4C). Linear models for leptin levels could not be built from data comparing weight-matched RC → HFD and HFD → RC animals (Table 1: p = 0.22).

#### Thyroid hormones

Using RNA-seq and RT-qPCR, we found *trh* expression in the VH was highest in HFD NoDS mice and intermediate in both DS conditions compared to RC NoDS animals (Fig 3J–3L). Since TRH initiates the hypothalamic-pituitary-thyroid (HPT) axis [36], we wondered whether circulating thyroid factors would exhibit a similar increase. We measured thyroid-stimulating hormone (TSH), triiodothyronine (T3), and thyroxine (T4) in the blood. Due to an unknown reason, TSH levels were highly variable between animals and thus limited interpretation of the results (Panel A in S4 Fig: p = 0.97). Further, 59% of samples could not measure plasma T3 above the detection threshold (not shown). However, T4 levels were robustly detected in all samples, but were statistically similar across all groups (Panel B in S4 Fig: p = 0.20). Since we were unable to reproduce known obesity-related HPT axis differences [37, 38], our interpretation of TSH, T3, and T4 measurements are limited.

### Alternate linear modeling approach to examine diet/energy balance effects independent from body weight

#### Linear models including all four groups can better estimate effects of ‘body weight’

In the current report, we used linear modeling to isolate ‘body weight’ and ‘diet/energy balance’ effects by comparing the two weight-matched DS groups, as previously described [11]. While this approach is able to parse apart their independent effects on each outcome metric, it is limited for two reasons. First, the model does not include data from RC NoDS and HFD NoDS groups, which provide valuable information about the physiological range of these metrics in obese animals and lean controls. Second, these models have difficulty quantifying associations with body weight because body weight is similar between RC → HFD and HFD → RC conditions. These shortcomings are evident, for example, when modeling plasma leptin levels (Fig 4C and Table 1). Despite the clear correlation between leptin levels and body weight (Fig 1C) across all four groups, linear models including only RC → HFD and HFD → RC conditions could not be built for leptin (Table 1: p = 0.22). To this end, we introduce a different linear modeling approach that includes data from all four conditions and assumes that ‘diet/energy balance’ can be approximated by acute weight changes (Δbody weight; % weight change from Pre- to Post-DS; see Fig 1D). We believe this is a valid assumption because HFD-fed obesity resistant rodents do not show stereotypical metabolic and behavioral abnormalities compared to HFD-fed obesity prone rodents [39–41], suggesting HFD-induced weight changes (not HFD *per se*) are required to induce pathophysiological conditions. Last, there is no correlation between body weight and Δbody weight among all animals (S5 Fig: R^2^ = 8.8x10^-5^, p = 0.94), and this variability helps to build statistically powerful linear associations for each output metric. The results for these new models are shown in Table 3, along with reanalyzed data from our previous manuscript, which focused on EEG-measured sleep/wake behavior following the same DS feeding paradigm [11].

**Table 3:** Linear modeling results using body weight and Δbody weight as predictors from all animals.

Metric	Linear Regression Models All four groups							
	ANOVA		Body Weight			ΔBody Weight		
	*F-value*	*p-value*	*β* *(SEM)*	*Stdβ*_*xy*_	*p-value*	*β* *(SEM)*	*Stdβ*_*xy*_	*p-value*
**Locomotor Mice**								
***Animal Behavior***								
**Caloric Int**. 24 Hour	**17.18**	**<0.0001**	0.04 (0.068)	0.059	0.56	**0.21** **(0.04)**	**0.583**	**<0.0001**
**Activity** Dark	**11.45**	**<0.0001**	**-894.60** **(188.35)**	**-0.504**	**<0.0001**	49.71 (96.34)	0.055	0.61
**Activity** Light	0.561	0.57	-86.47 (82.85)	-0.127	0.30	7.02 (42.38)	0.020	0.87
**Activity** 24 Hour	**9.46**	**0.0002**	**-981.07** **(227.38)**	**-0.468**	**0.0001**	56.73 (116.30)	0.053	0.63
***VH Gene Expression***								
***Agrp***	**15.98**	**<0.0001**	**-0.066** **(0.017)**	**-0.444**	**0.0003**	**-0.037** **(0.008)**	**-0.496**	**0.0001**
***Npy***	**21.79**	**<0.0001**	**-0.067** **(0.015)**	**-0.469**	**0.0001**	**-0.040** **(0.008)**	**-0.553**	**<0.0001**
***Cartpt***	**7.22**	**0.0019**	**0.047** **(0.012)**	**0.489**	**0.0004**	0.003 (0.006)	0.072	0.58
***Pomc***	1.92	0.16	0.017 (0.018)	0.133	0.0004	0.016 (0.009)	0.254	0.081
***Trh***	**6.29**	**0.0038**	**0.057** **(0.016)**	**0.463**	**0.0009**	-0.001 (0.008)	-0.008	0.95
***Sbno2***	**4.14**	**0.022**	**0.023** **(0.009)**	**0.353**	**0.010**	-0.005 (0.005)	-0.142	0.30
***Serpina3n***	**7.66**	**0.0013**	**0.045** **(0.012)**	**0.494**	**0.0004**	-0.002 (0.006)	-0.046	0.72
***Blood Biomarkers***								
**Glucose**	**40.73**	**<0.0001**	**3.72** **(0.72)**	**0.427**	**<0.0001**	**2.76** **(0.37)**	**0.609**	**<0.0001**
**Insulin**	**34.26**	**<0.0001**	**331.44** **(43.07)**	**0.701**	**<0.0001**	**84.26** **(25.92)**	**0.296**	**0.0020**
**Leptin**	**10.68**	**0.0001**	**268.09** **(60.21)**	**0.516**	**<0.0001**	45.49 (35.68)	0.148	0.21
**T4**	2.41	0.10	**-3629** **(1655)**	**-0.288**	**0.033**	61.95 (980.6)	0.008	0.95
**EEG Mice: Behavior**								
***Caloric Intake***								
**Caloric Int**. 24 Hour	0.461	0.64	-0.050 (0.073)	-0.145	0.50	0.035 (0.042)	0.175	0.42
***Total Time Spent in Each State***								
**Wake** 24 Hour	**13.38**	**0.0001**	**-6.50** **(1.99)**	**-0.481**	**0.003**	**-3.47** **(1.15)**	**-0.443**	**0.006**
**NREM** 24 Hour	**11.33**	**0.0004**	**6.04** **(1.93)**	**0.480**	**0.005**	**2.97** **(1.12)**	**0.406**	**0.014**
**REM** 24 Hour	**3.68**	**0.041**	0.461 (0.416)	0.209	0.28	**0.506** **(0.241)**	**0.395**	**0.047**

Estimates are from joint regression models containing both weight factors. ANOVA: Indicates overall significance of linear model. *β*: β-estimate and standard error indicates expected change in outcome for a 1 unit increase in predictor. *Stdβ*_*xy*_: Standardized β-estimate along both axes, indicating the standard deviation change for the y-variable given a 1 standard deviation increase in the predictor. SEM: Standard Error of the Mean. Highlighted cells indicate significance at p<0.05. Note that VH gene expression values are modeled using RT-qPCR results from sequenced tissue of individual animals (Fig 3 and S2 Fig: middle rows) to maximize sample size. REM: Rapid eye movement sleep; NREM: Non-REM sleep; *cartpt*: cocaine- and amphetamine regulated transcript; *serpina3n*: serine (or cysteine) peptidase inhibitor clade A member 3N; *sbno2*: strawberry notch homolog 2; *trh*: thyrotropin-releasing hormone; *agrp*: agouti-related peptide; *npy*: neuropeptide y; *pomc*: proopiomelanocortin; T4: Thyroxine.

#### Reanalyzing previous results (EEG mice) to compare behavioral responses in the present study

In the present study, caloric intake is positively related to Δbody weight (Table 3: p<0.0001) but not actual body mass (p = 0.56; visualized in Figures A and B in S6 Fig), which is similar to the results of the previous model showing an association with diet/energy balance (Table 1: p<0.0001) but not body weight (p = 0.35). Therefore, significant associations with ‘Δbody weight’ may be mediated, in part, by caloric intake differences, which is similar to the ‘diet/energy balance’ predictor in the previous model. Regarding the EEG-mice, caloric intake was similar across all conditions on Day 7 Post-DS [11], and statistically significant models for energy intake could not be built (Table 3: p = 0.64). Thus, unexpected caloric intake differences between the previous and current studies following the DS feeding paradigm may be due to housing conditions (tethered EEG recording cables vs homecage locomotor boxes).

Next, we reanalyzed how body weight and Δbody weight are associated with EEG-measured sleep/wake behavior and compared those results to the locomotor activity data presented herein. We found that total wake and non-rapid eye movement sleep (Non-REM, or NREM) time were significantly associated with both body weight (Table 3: p = 0.003 and p = 0.005, respectively) and Δbody weight (p = 0.006 and p = 0.014, respectively); REM sleep was positively associated with Δbody weight (p = 0.047) but not body mass (p = 0.28). However, in the present study, spontaneous activity is negatively correlated with body mass (p = 0.0001) but is unrelated to Δbody weight (p = 0.63; visualized in Panels C and D in S6 Fig); this effect is largely driven by the obese, hypoactive HFD NoDS mice (Fig 2C). Taken together, these linear models highlight how body mass and acute weight changes differentially affect spontaneous locomotor and sleep/wake behavior, and provide better estimates for body weight effects compared to the models used earlier.

#### Relating blood metrics and VH gene expression to activity and sleep/wake behavior

**Fig 5:**
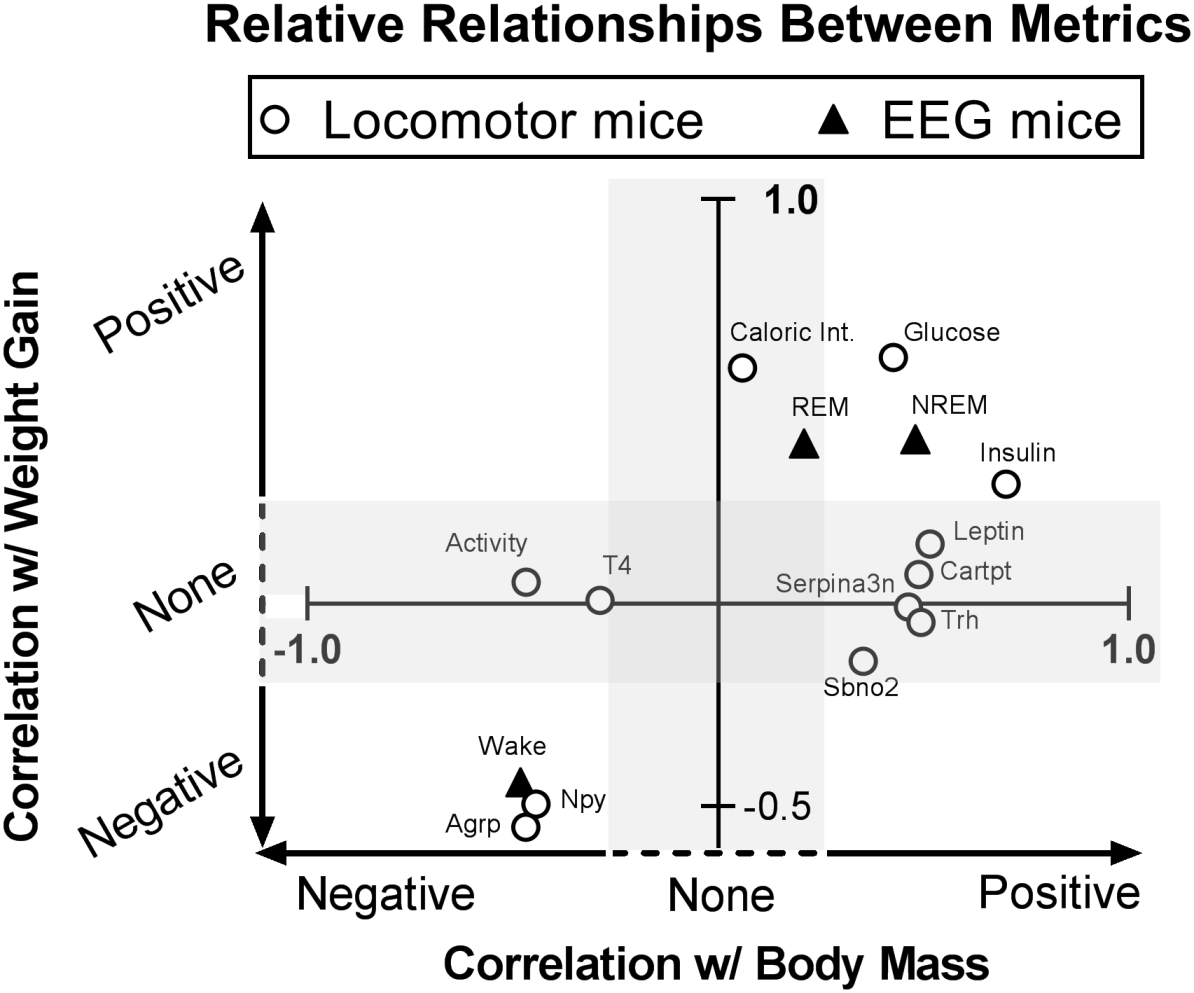
Plotting standardized correlation coefficients to visualize how blood biomarkers, VH gene expression, and behavioral data relate. For each output metric, standardized β coefficients (Stdβ_xy_) were calculated for both weight metrics (body weight and Δbody weight) and plotted together here. Any values to the right of the y-axis are positively associated with increased body weight, while any values to the left of the y-axis decrease as body mass increases. Any values above the x-axis increase as Δbody weight increases (positive energy balance), while points below the x-axis decrease as Δbody weight increases. Points in the shaded area represent non-significant associations. Exact Stdβ_xy_ and p-values are shown in Table 3. REM: Rapid eye movement sleep; NREM: Non-REM sleep; *cartpt*: cocaine- and amphetamine regulated transcript; *serpina3n*: serine (or cysteine) peptidase inhibitor clade A member 3N; *sbno2*: strawberry notch homolog 2; *trh*: thyrotropin-releasing hormone; *agrp*: agouti-related peptide; *npy*: neuropeptide y; T4: Thyroxine.

To this point, we have calculated how each metric relates to both body mass and Δbody weight by using the DS feeding paradigm. Specifically, the relationship of each output measure to both predictors (body weight and Δbody weight) was described by β-values, or slope of the linear fit. In general, if a predictor and outcome are strongly related, the magnitude of the slope will increase (and the p-value will decrease). In order to compare these β-values directly, both the x and y distributions (mean ± standard deviation) were normalized to Z-scores. This yields a standardized score along both axes (Stdβ_xy_) that can now be interpreted as the magnitude increase (in standard deviations) of the output variable given a 1 standard deviation increase in the predictor variable. Therefore, we calculated two Stdβ_xy_ scores for each metric (one each for its relation to body weight and Δbody weight), and plotted these values in Fig 5.

We found that the data can be generally categorized into five groups. First, some metrics are positively related to body weight without a relation to Δbody weight (plasma leptin and VH expression of *cartpt*, *trh*, *sbno2*, *serpina3n*). Second, two metrics—total spontaneous activity and plasma T4 levels—are negatively associated with body weight and display no relationship to Δbody weight (although the linear model for T4 only trends towards significance; Table 3: p = 0.10). Third, caloric intake and total REM sleep are positively related to Δbody weight and unrelated to body weight. Fourth, some metrics are positively associated with both increased body weight and acute weight gain, including plasma glucose and insulin and total NREM sleep time. Last, total wakefulness and VH expression of *agrp* and *npy* are negatively associated with both increased body weight and weight gain. Altogether, this approach allows visualization of correlated metabolic and behavioral parameters which helps to inform how peripheral and central processes may influence spontaneous activity and sleep/wake patterns.

## Discussion

Using the DS feeding paradigm, we generated two groups of mice (RC → HFD and HFD → RC) with similar body mass but opposite energetic status. Using two different linear modeling approaches, we found that VH expression of *agrp* and *npy*, blood glucose and insulin levels, and sleep/wake behavior were highly modified by diet and/or energy balance. Unlike total wakefulness, spontaneous locomotor activity was not related to diet/energy balance; VH expression of *cartpt*, *trh*, *sbno2*, and *serpina3n* as well as plasma leptin and T4 show similar associations to activity patterns. Taken together, our results reveal novel associations between blood biomarkers, neural gene expression, and spontaneous activity and sleep/wake behavior, which offers new insights into the genesis and reversibility of metabolic and behavioral abnormalities in DIO.

DIO animals are both profoundly hypoactive and hypersomnolent, spending an extra 1–2 hours asleep per day compared to lean controls [23]. Spontaneous activity and sleep/wake patterns are highly correlated, since inactivity is a requirement for sleep. However, we found that wakefulness and NREM sleep, but not spontaneous locomotor activity, were significantly associated with Δbody weight (Table 3). These differences may be related to technical limitations of the locomotor boxes or a real biological phenomenon. Regarding the former, locomotor boxes track activity using infrared beams; when a beam is ‘broken’ by a moving animal, the software registers an activity count. Since obese animals are physically wider, they will be able to break more beams with less movement compared to lean mice. Other activity monitoring approaches, such as video tracking, may provide a less biased approach to compare lean vs obese animals’ activity patterns [42]. Second, the difference between locomotor activity and wakefulness may be a natural phenomenon. Specifically, our previous work found that the RC → HFD animals exhibit abnormal sleep behavior, with similar sleep/wake amounts and fragmentation to HFD NoDS animals [11]. However, in the present study, RC → HFD animals were hyperactive compared to HFD NoDS mice (Fig 2C: p<0.001). One interpretation is that RC → HFD animals increase physical activity to counteract weight gain, but this leads to physical exhaustion, fatigue, and sleepiness. Indeed, obesity is associated with decreased endurance and increased fatigue [43]. Furthermore, HFD-fed animals consume food throughout the day [44], which may cause increased activity acutely (while eating) but post-prandial rest immediately afterwards [45]. Conversely, animals losing weight (HFD → RC) show similar wake and activity patterns to RC NoDS animals, suggesting that the mechanisms that induce hypersomnolence may be separate from the mechanisms that rescue it. Lastly, REM sleep was positively associated with Δbody weight, but not absolute body weight (Table 3). However, REM sleep amounts are highly inconsistent between different mouse models of obesity [23], so it remains unclear how diet, energy balance, and body weight relate to REM sleep. Future work that measures activity and sleep/wake behaviors simultaneously will be required to confirm their uncoupling under these conditions. However, when we attempted to measure HFD-induced changes to both EEG-recorded sleep/wake and infrared beam-based locomotor activity, mice did not experience normal weight gain. We believe this was due to the combined stressors required for each signal acquisition (tether cables for EEG; low bedding and no nestlet for locomotor activity). This further highlights the need for advanced simultaneous tracking of both behaviors (e.g., telemetry-based EEG recordings with video tracking).

The present study used RNA-seq to discover VH genes and pathways which were differentially affected by body weight and/or diet/energy balance. Of the short list of DEGs, many genes encoded neuropeptides with known roles in energy balance, locomotor activity, and sleep/wake behavior. Specifically, we detected thyrotropin-releasing hormone (*trh*) as gene of interest. Since *trh* initiates the hypothalamus-pituitary-thyroid (HPT) axis, we measured thyroid hormone levels in the blood, but an unknown source of variability in TSH and T3 measurements limited the overall interpretation of our results. Nonetheless, VH expression of *trh* and blood T4 correlated with body mass independent of Δbody weight, although *trh* was positively associated with body weight while T4 was negatively associated. Similar trends are observed in both sleep disturbed and obese animals, including obese humans losing weight via caloric restriction [38, 46, 47]; however, since sleep differences were controlled for prior to sacrifice, we believe these associations with body weight are not confounded by behavioral differences. Thus, our work suggests that obesity-related HPT dysfunctions are related to body weight independent of acute diet or weight changes and sleep/wake differences, although reliable TSH and T3 measurements are still required.

In addition to *trh*, neuropeptide y (*npy*), agouti-related peptide (*agrp*), and cocaine- and amphetamine regulated transcript (*cartpt*) were all found to be differentially expressed by RNA-seq. All three genes are enriched in the ARC with well-known roles in maintaining energy balance [18]. Furthermore, obese animals have decreased *npy* and *agrp* expression and increased *cartpt* expression [21, 32]. Here, we show that *npy* and *agrp* are not only decreased in obese (HFD NoDS) animals, but also in HFD → RC animals; therefore, *npy* and *agrp* are negatively associated with both increased body mass and acute weight gain. However, while *cartpt* is positively related to body weight, it is not affected by Δbody weight. These observations are interesting for a few reasons. First, there is a negative association between VH gene expression and known downstream effects of the encoded neuropeptide. For example, HFD NoDS animals have augmented expression of *cartpt*, but suffer from the hypersomnolence and fragmented sleep/wake bouts [23]. However, exogenous CARTPT neuropeptide potently increases wakefulness and consolidates sleep/wake bouts [48]. Therefore, it is possible that endogenous CARTPT levels are decreased (despite elevated transcript levels) and/or activation of downstream signaling pathways are blunted. Indeed, resistance to anorexigenic signaling pathways are well-characterized in obesity (e.g., leptin and insulin), including neuropeptides such as CARTPT [49]. Second, out of all of VH genes measured, only genes that promote food intake (*agrp* and *npy*) responded to acute body weight changes. While it remains unclear if this has a functional consequence, this suggests that the VH senses and responds to weight loss by increasing expression of orexigenic genes, but during weight gain, anorexigenic gene expression does not immediately elevate. This asymmetry between anorexigenic and orexigenic pathways would also perpetuate weight gain. Taken together, we recapitulate known associations with *npy*, *agrp*, and *cartpt* with obesity, but show that only the orexigenic genes (*npy* and *agrp)* are related to acute weight changes.

RNA-seq has the advantage of quantifying all mRNA transcripts in the tissue sample, revealing understudied genes or pathways beyond putative VH neuropeptides. First, RNA-seq analysis revealed upregulation of *serpina3n* and *sbno2* in HFD NoDS animals, demonstrating a positive correlation with body weight with no relation to Δbody weight. *Sbno2* is a pro-inflammatory factor in the CNS expressed predominately in astrocytes [50], while se*rpina3n* is an astrogliosis marker with elevated expression in the hypothalamus of DIO mice [51, 52]. Obesity is known to induce hypothalamic inflammation [53, 54], and reducing this inflammation can protect against HFD-induced weight gain [55, 56]. While we did not directly quantify astrogliosis and VH inflammation in our studies, we found that these inflammatory markers do not increase acutely and are only elevated in DIO mice. Future work directly measuring hypothalamic inflammation (via gene and protein expression assays of many biomarkers) will be required to fully establish the link to diet/energy balance. Second, we ran RNA-seq results through GSEA to discover biochemical pathways that may differ between DS groups. While this approach is prone to false-positives, ‘Oxidative Phosphorylation’ was identified twice as significantly enriched in HFD → RC animals compared to RC → HFD (S3 Table: p<0.0001). This potentially implicates differential energy production between weight-matched DS animals, which could be due to alternate sources of energy (e.g., anaerobic pathways) or indicate differences in mitochondrial functioning. Regarding the latter, obesity has known associations with abnormal mitochondrial biogenesis, morphology, and ATP production [57]. However, the influence of diet and/or energy balance on mitochondria, independent of body weight, remains unclear. Taken together, RNA-seq is a powerful hypothesis-generating tool that provides unbiased expression of all transcripts in a given tissue sample.

RNA-seq, however, does have limitations, especially when measuring heterogeneous tissues such as the brain. Like other gene expression assays (microarrays and RT-qPCR), RNA-seq is most powerful with homogenous samples [58]. The VH, however, contains many neural cell types (e.g., neurons, astrocytes, microglia), distinct neuronal nuclei (e.g., ARC, PVN, and VMN), and the ARC alone contains as many as 50 transcriptionally-distinct cell types [59]. Thus, assessing gene expression changes in VH punches will dilute relevant ‘signal’ with the ‘noise’ of other cell types. Indeed, RNA-seq of purified p*omc*+ and *agrp*+ ARC neurons from lean, *ad lib*-fed mice found hundreds of DEG [60]. However, this purification process requires 3+ hours of cell manipulation before cell lysis and RNA extraction, which may have unintended effects on expression profiles. Importantly, our approach did identify genes with robust expression differences, which was confirmed using RT-qPCR of tissue samples from an independent animal cohort.

In addition to the VH, the brainstem contains nuclei critical for sensing and integrating peripheral and central signals to influence metabolic and behavioral phenotypes. Specifically, the dorsal vagal complex (DVC) is a heterogeneous hindbrain region that responds to changes in blood glucose, insulin, and leptin, and projects to many other brain regions (including the hypothalamus) to regulate food intake [61–64]. Furthermore, many gut-derived hormones (such as cholecystokinin and glucagon-like peptide-1) can reduce food intake by acting on the DVC directly (via receptor activation) or indirectly (via vagal afferents) [12, 64]. Future work could measure how other gut-derived hormones and brain regions (specifically the DVC) respond to changes in diet/energy balance vs body weight.

Gene expression assays (such as RNA-seq and RT-qPCR) do not provide information about downstream processes (e.g., protein translation/cleavage/degradation), which may be uncoupled from mRNA expression levels. For example, hypothalamic *pomc* transcript levels are unchanged in DIO [32, 33], but the encoded POMC peptide is decreased in obese rats [65]. Furthermore, POMC is cleaved into multiple peptide hormones (e.g., adrenocorticotropic hormone and α-melanocyte-stimulating hormone), and individual hormones can be affected by fasting/overfeeding and directly influence metabolic and behavioral phenotypes [18, 66–68]. Future studies measuring neuropeptide and hormone levels following the DS feeding paradigm may implicate other signaling pathways not identified by RNA-seq.

The present study uses two linear regression approaches to determine independent ‘body weight’ and ‘diet/energy balance’ effects on measured parameters. The first model only uses data from the two weight-matched DS groups (RC → HFD and HFD → RC) and uses an arbitrary categorical variable as the ‘diet/energy balance’ predictor. By modeling ‘diet/energy balance’ with a categorical variable, all confounding factors between with the different DS groups (e.g., caloric intake, macronutrient composition, energy balance, etc.) are included in this sole variable. Therefore, this model can isolate ‘body weight’ effects independent of these other factors. However, this approach does have its disadvantages, particularly when quantifying associations that appear to correlate with ‘body weight’ (e.g., leptin levels). To this end, we used a second modeling approach that estimates ‘diet/energy balance’ with acute weight changes (Δbody weight). This has a few unique advantages. First, the DS feeding paradigm generates large variability between groups for both body weight (min / median / max:: 26.5 / 33.8 / 50.7; in grams) and Δbody weight (-20.5 / 1.70 / 22.0; in %) which is normally distributed around the mean. Second, the animals with the most extreme body weight differences (RC NoDS and HFD NoDS) have the smallest changes in Δbody weight, and vice versa for the DS groups. Third, this approach uses real data for all input and output metrics, as opposed to the arbitrary categorical values, thus capturing interanimal variability associated with RC and HFD consumption. Last, since all animals are included in the analysis, estimated β-coefficients for body weight are more powerful, and standardized β-coefficients *(Stdβ*_*xy*_*)* for body weight and Δbody weight can be directly compared. To this end, we scatter-plotted the calculated *Stdβ*_*xy*_ values for each metric together, allowing for a snapshot of how blood biomarkers, VH gene expression, and behavior correlate, which revealed novel associations between these parameters.

The main drawback to this linear modeling approach, however, is that it assumes the majority of diet-induced metabolic effects are due to weight gain/loss. Therefore, this model cannot capture pure diet effects (due to differences macronutrient composition) or differences in caloric intake. In order to fully disassociate ‘diet’ from ‘energy balance’, future work could repeat the DS with different hypercaloric diets (that vary by carbohydrate, fat, and protein content), alter the duration of post-DS exposure (both less than and greater than one week), and/or use other approaches to affect acute weight changes (e.g., access to a running wheel). In addition, future studies that measure basal metabolic rate and energy expenditure may help disassociate ‘diet’ and ‘energy balance’ effects on metabolic and behavioral outcomes. Finally, this modeling approach may overestimate body weight effects, since RC NoDS and HFD NoDS conditions have the most extreme body weight and metabolic/behavioral differences. Indeed, longer exposure to HFD would further augment body mass, thus changing the linear model coefficient estimates or the overall association (e.g., logarithmic relationship) between body weight and the outcome of interest. Nonetheless, we believe this modeling approach succinctly estimates trends across all groups, allowing each output metric to be compared to one another.

It is important to acknowledge the limitations of this research. First, we did not quantify amount of lean and fat mass in these mice. Obesity is typically classified by BMI because it is easy to measure, but visceral abdominal fat is a better predictor for long term health outcomes in humans [69, 70]. In mice, previous studies have found HFD greatly increases both fat mass and body weight, with little to no effect on lean mass, and that body weight correlates with fat mass (but not lean mass) after DIO mice are switched to RC (HFD → RC) [71, 72]. Therefore, since body weight and fat mass correlate following RC and HFD challenges, we believe building linear models based on body fat would produce similar associations to outcome metrics (compared to body weight). Furthermore, since fat and lean mass is typically quantified either by dual energy X-ray absorptiometry (which requires anesthesia) or post-mortem in mice [69], it would be difficult to quantify within-animal changes from Pre- to Post-DS, which was an important predictor in the linear models. Nonetheless, relating these metrics to fat mass instead of body mass may reveal some unexpected and interesting differences. Second, we chose to sleep deprive, but not fast mice, prior to sacrifice. Sleep deprivation controlled for known differences in behavior between lean and obese mice [11, 23], allowing us to measure VH genes that may be upstream of sleep/wake changes. Furthermore, we chose to maintain *ad lib* feeding conditions to prevent all animals from losing weight prior to sacrifice. Indeed, fasting normally-weighted mice can reduce lean mass and result in torpor, which is characterized by decreased metabolic rate, hypoactivity, and hypothermia [73, 74]; therefore, we wanted to avoid these potential confounds. Importantly, we found no evidence of torpor in our studies, since spontaneous activity levels were elevated in all groups (including HFD → RC animals, which were rapidly losing weight) compared to HFD NoDS animals. While we acknowledge that random feeding increases variability in VH genes and blood biomarkers compared to fasting [73, 75], we still found robust differences among these metrics (e.g., VH *agrp*, *npy*, *cartpt*, *trh*; blood glucose, insulin, leptin) which had unique expression patterns from one another. Therefore, it is unlikely that random feeding greatly influenced these group-wide differences, although it is possible fasting would further augment these effects.

Collectively, our results reveal how distinct aspects of DIO (diet/energy balance and body weight) can independently affect many metabolic and behavioral outcomes. We found many metrics were highly modified by changes to diet/energy balance, including glucose and insulin, VH expression of *npy* and *agrp*, and sleep/wake behavior. This suggests that these parameters are more related to one another compared to factors with no association with diet/energy balance. Furthermore, the physiological changes which are modified by diet/energy balance necessarily precede changes associated with body weight. For example, even though DIO mice are both hypersomnolent and hypoactive, RC → HFD animals are profoundly hypersomnolent but paradoxically hyperactive one week Post-DS [11]. Therefore, our work reveals that obesity-related sleep/wake abnormalities present rapidly (within 1 week) while locomotor activity differences require longer to manifest. Identifying the directionality of obesity-related changes (e.g., sleep/wake changes are upstream of activity changes) may help elucidate the genesis and reversibility of DIO comorbidities.

## Supporting information

S1 Fig Activity-based estimates for sleep/wake behavior are unreliable for DIO mice. Obese mice sleep ~1–2 hours more per day and exhibit increased sleep/wake fragmentation compared to lean animals [23]. Fig 2A shows that DIO mice are hypoactive compared to lean mice during the dark period, when these sleep effects are most pronounced. An activity-based algorithm to estimate wake differences has been developed and validated in lean mice; this ‘40-second’ rule agrees >90% with simultaneous EEG-recordings [22]. We find that this algorithm does not recapitulate stereotypical sleep/wake abnormalities observed in DIO mice (C,G). Further, alternate algorithms using (A,E) 20, (B,F) 30, or (D,H) 50 seconds of inactivity as a cut-off threshold for sleep shows similar inadequacies. RC NoDS, n = 16; RC → HFD, n = 21; HFD → RC, n = 19; HFD NoDS, n = 14.
(TIF)

S2 Fig RT-qPCR validation of RNA-seq results including neuropeptide genes for which differential expression was not expected. (Top row) Normalized read counts from RNA-seq analysis. (Middle row) RT-qPCR validation using tissue used for RNA-seq. (Bottom row) RT-qPCR validation using independent tissue samples not used for RNA-seq. (A-C) *Sbno2* and (D-F) *serpina3n* showed highest expression level in HFD NoDS, lowest in RC NoDS, and low to intermediate expression in both DS groups. (G-I) *Pomc* and (J-L) *hcrt* expression was similar across all groups, as expected. *serpina3n*: serine (or cysteine) peptidase inhibitor clade A member 3N; *sbno2*: strawberry notch homolog; *pomc*: proopiomelanocortin; *hcrt*: hypocretin/orexin. a’: p<0.01, a”: p<0.001, a”‘: p<0.0001 compared to RC NoDS; b: p<0.05, b’: p<0.01, b”: p<0.001, b”‘: p<0.0001 compared to HFD NoDS. Sample sizes for top row: [RC NoDS, n = 5; RC → HFD, n = 6; HFD → RC, n = 5; HFD NoDS, n = 5]. Sample sizes for middle row: [RC NoDS: n = 13; RC → HFD: n = 18; HFD → RC: n = 11; HFD NoDS: n = 7]. Samples sizes for bottom row: [RC NoDS: n = 5; RC → HFD: n = 8; HFD → RC: n = 6; HFD NoDS: n = 3].
(TIF)

S3 Fig RNA-seq results of remaining DEGs discovered by any RNA-seq analysis approach (see Table 1). Note the low expression counts for *izfk3*, *tk1*, and *cldn2*. *Ikfz3*: IKAROS family zinc finger 3; *tk1*: thymidine kinase 1; *cldn2*: claudin 2; *atxn7l2*: ataxin 7 like 2; *rbm3*: RNA-binding protein 3; *c4b*: complement 4b. **p<0.01, ***p<0.001 comparing two DS conditions; a: p<0.05, a’: p<0.01, a”: p<0.001, a”‘: p<0.0001 compared to RC NoDS; b: p<0.05, b’: p<0.01, b”: p<0.001, b”‘: p<0.0001 compared to HFD NoDS. RC NoDS, n = 5; RC → HFD, n = 6; HFD → RC, n = 5; HFD NoDS, n = 5.
(TIF)

S4 Fig Thyroid hormone measurements. (A) Thyroid-stimulating hormone (TSH) showed highly variable expression levels across samples, contributing to non-significant group effects (p = 0.9658). (B) Thyroxine (T4) levels were not robustly different between dietary conditions. Triiodothyronine (T3) levels were also measured, but most samples were below detection threshold and not quantifiable (not shown). TSH sample sizes: [RC NoDS, n = 11; RC → HFD, n = 14; HFD → RC, n = 11; HFD NoDS, n = 11]. T4 sample sizes: [RC NoDS, n = 13; RC → HFD, n = 17; HFD → RC, n = 13; HFD NoDS, n = 13].
(TIF)

S5 Fig Body weight and Δbody weight are not correlated across all dietary conditions. Scatter plot of absolute body weight (Post-DS, in grams) vs Δbody weight (percent change in body weight from Pre- to Post-DS). There is no correlation between body weight and Δbody weight for these mice across all groups (R^2^ = 8.8x10^-5^, p = 0.94). RC NoDS, n = 16; RC → HFD, n = 21; HFD → RC, n = 19; HFD NoDS, n = 14.
(TIF)

S6 Fig Linear regression analysis for caloric intake and locomotor activity. (A) Caloric intake is not related to body weight. (B) Energy intake is positively related to acute weight changes (Δbody weight). (C) Activity patterns are negatively associated with body weight. (D) Locomotor activity is not related to Δbody weight.
(TIF)

S7 Fig Immunofluorescence localization of the VH. One mouse was deeply anesthetized and transcardially perfused with ice-cold 4% paraformaldehyde (pH = 7.5). The brain was left in 4% PF overnight, and then switched to 30% sucrose the following day. A cryostat was used to coronally section the brain (50 μm) and free-floating sections were placed into blocking buffer (4% normal donkey serum and 0.4% Triton-X in 1x PBS) overnight. The following day, anti-NeuN (1:1000, MAB377, EMD Millipore) was diluted in blocking buffer. Brain slices were incubated in primary antibody at 4°C on a shaker for 3 days. The primary was then washed off thrice with 1X PBS, and the secondary antibodies (A21206, Invitrogen, Carlsbad, CA) was applied (1:500 in 50% blocking buffer:50% 1X PBS) for 1 hour. Image was taken at 20x magnification.
(TIF)

S1 Table RNA-seq total read counts and contamination from ribosomal RNA and mitochondrial DNA. (DOCX)

S2 Table Gene set enrichment analysis (GSEA) results. Using the ‘High Filter’ data set of RNA-seq results (see Methods), GSEA reveals significantly enriched pathways in HFD → RC animals compared to RC → HFD. Using both raw and log2-transformed data, we found the same six enriched pathways at an FWER < 0.05. FDR: False discovery rate; FWER: Family wise error rate; PPARg: Peroxisome proliferator-activated receptor gamma.
(DOCX)

S3 Table Gene name, NCBI reference sequence, and catalog number of Taqman primer/probes used for RT-qPCR experiments. (DOCX)

S4 Table Details statistical results reporting adjusted p-values. Only comparisons that reached overall significance (ANOVA: p<0.05) are shown.
(DOCX)
