# Supplementary figures and images for: Dietary challenges differentially affect activity and sleep/wake behavior in mus musculus: Isolating independent associations with diet/energy balance and body weight
Source: PLoS One. 2018 May 10;13(5):e0196743. doi: 10.1371/journal.pone.0196743 (PMC5945034; doi:10.1371/journal.pone.0196743)

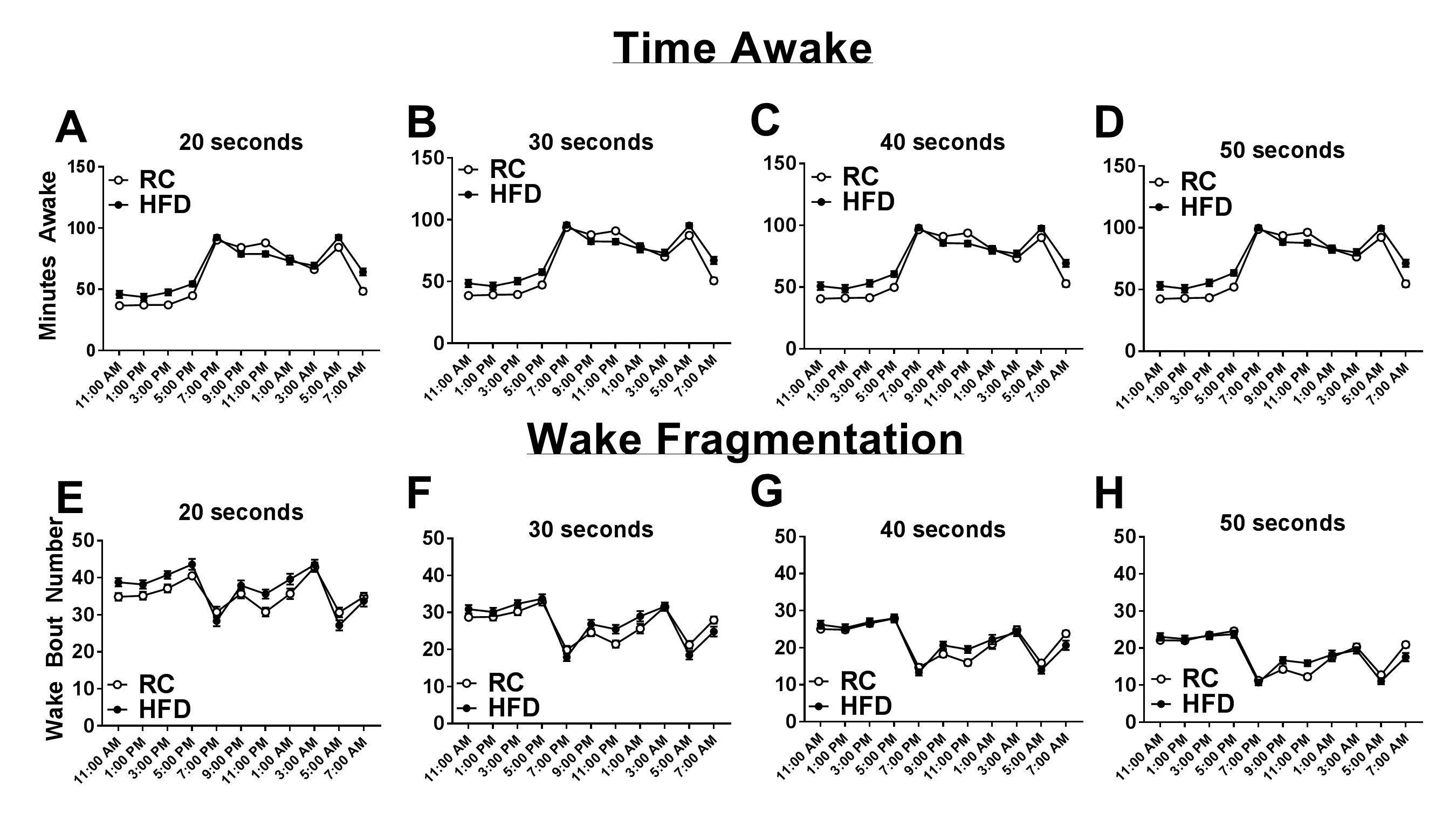

Supplement: S1 Fig — Obese mice sleep ~1–2 hours more per day and exhibit increased sleep/wake fragmentation compared to lean animals [23]. Fig 2A shows that DIO mice are hypoactive compared to lean mice during the dark period, when these sleep effects are most pronounced. An activity-based algorithm to estimate wake differences has been developed and validated in lean mice; this ‘40-second’ rule agrees >90% with simultaneous EEG-recordings [22]. We find that this algorithm does not recapitulate stereotypical sleep/wake abnormalities observed in DIO mice (C,G). Further, alternate algorithms using (A,E) 20, (B,F) 30, or (D,H) 50 seconds of inactivity as a cut-off threshold for sleep shows similar inadequacies. RC NoDS, n = 16; RC → HFD, n = 21; HFD → RC, n = 19; HFD NoDS, n = 14. (TIF) [file pone.0196743.s001.tif]

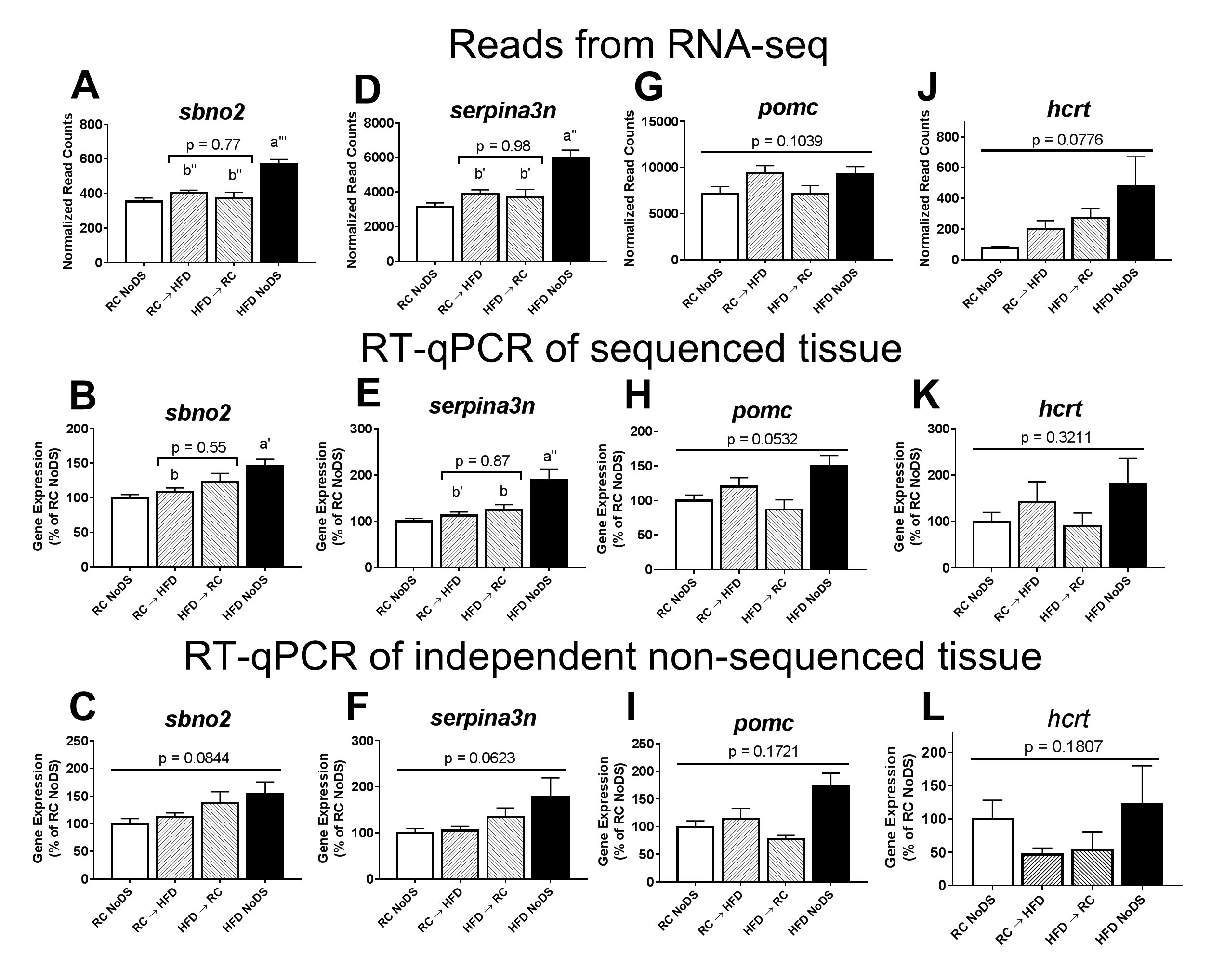

Supplement: S2 Fig — (Top row) Normalized read counts from RNA-seq analysis. (Middle row) RT-qPCR validation using tissue used for RNA-seq. (Bottom row) RT-qPCR validation using independent tissue samples not used for RNA-seq. (A-C) Sbno2 and (D-F) serpina3n showed highest expression level in HFD NoDS, lowest in RC NoDS, and low to intermediate expression in both DS groups. (G-I) Pomc and (J-L) hcrt expression was similar across all groups, as expected. serpina3n: serine (or cysteine) peptidase inhibitor clade A member 3N; sbno2: strawberry notch homolog; pomc: proopiomelanocortin; hcrt: hypocretin/orexin. a’: p<0.01, a”: p<0.001, a”‘: p<0.0001 compared to RC NoDS; b: p<0.05, b’: p<0.01, b”: p<0.001, b”‘: p<0.0001 compared to HFD NoDS. Sample sizes for top row: [RC NoDS, n = 5; RC → HFD, n = 6; HFD → RC, n = 5; HFD NoDS, n = 5]. Sample sizes for middle row: [RC NoDS: n = 13; RC → HFD: n = 18; HFD → RC: n = 11; HFD NoDS: n = 7]. Samples sizes for bottom row: [RC NoDS: n = 5; RC → HFD: n = 8; HFD → RC: n = 6; HFD NoDS: n = 3]. (TIF) [file pone.0196743.s002.tif]

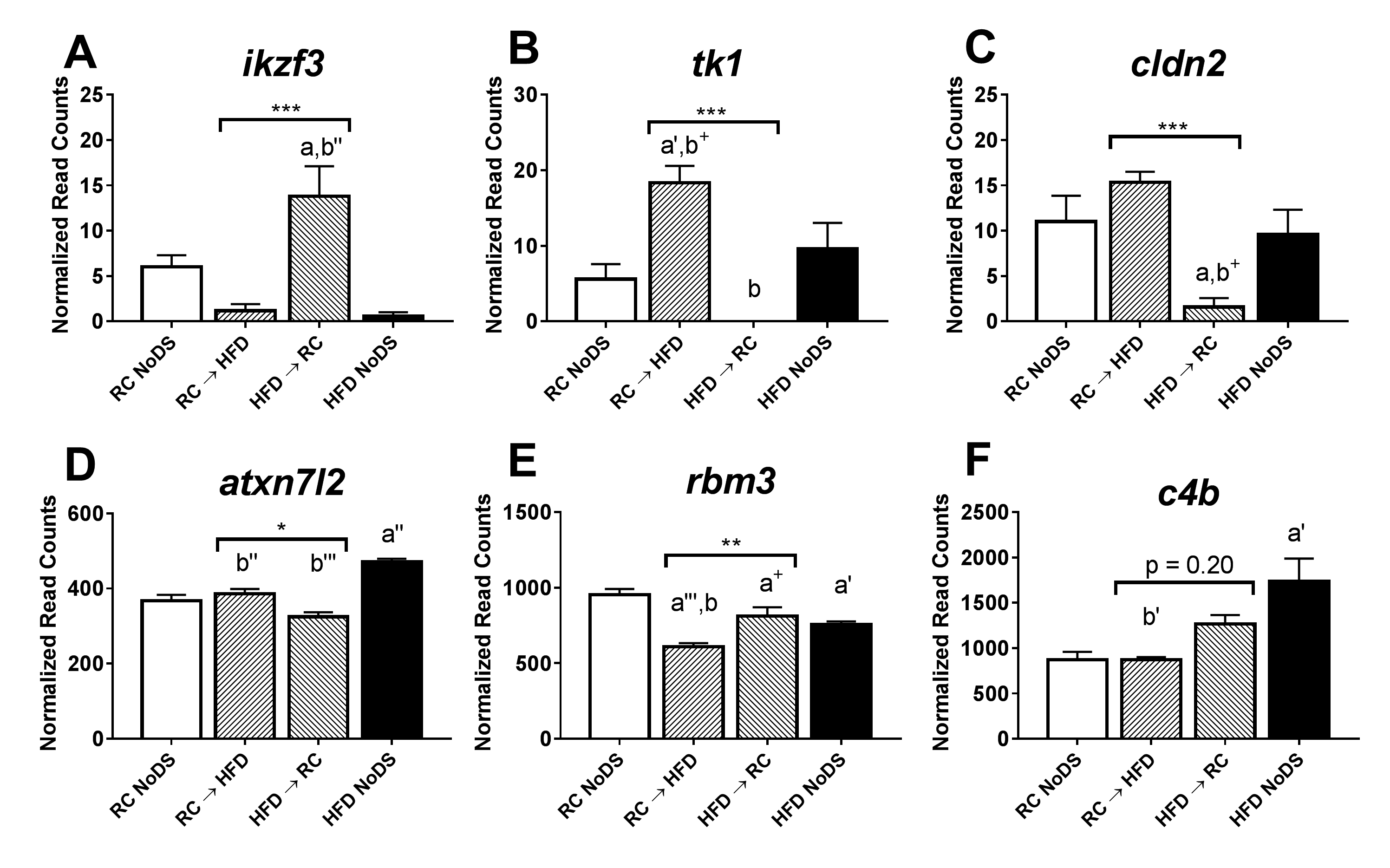

Supplement: S3 Fig — Note the low expression counts for izfk3, tk1, and cldn2. Ikfz3: IKAROS family zinc finger 3; tk1: thymidine kinase 1; cldn2: claudin 2; atxn7l2: ataxin 7 like 2; rbm3: RNA-binding protein 3; c4b: complement 4b. **p<0.01, ***p<0.001 comparing two DS conditions; a: p<0.05, a’: p<0.01, a”: p<0.001, a”‘: p<0.0001 compared to RC NoDS; b: p<0.05, b’: p<0.01, b”: p<0.001, b”‘: p<0.0001 compared to HFD NoDS. RC NoDS, n = 5; RC → HFD, n = 6; HFD → RC, n = 5; HFD NoDS, n = 5. (TIF) [file pone.0196743.s003.tif]

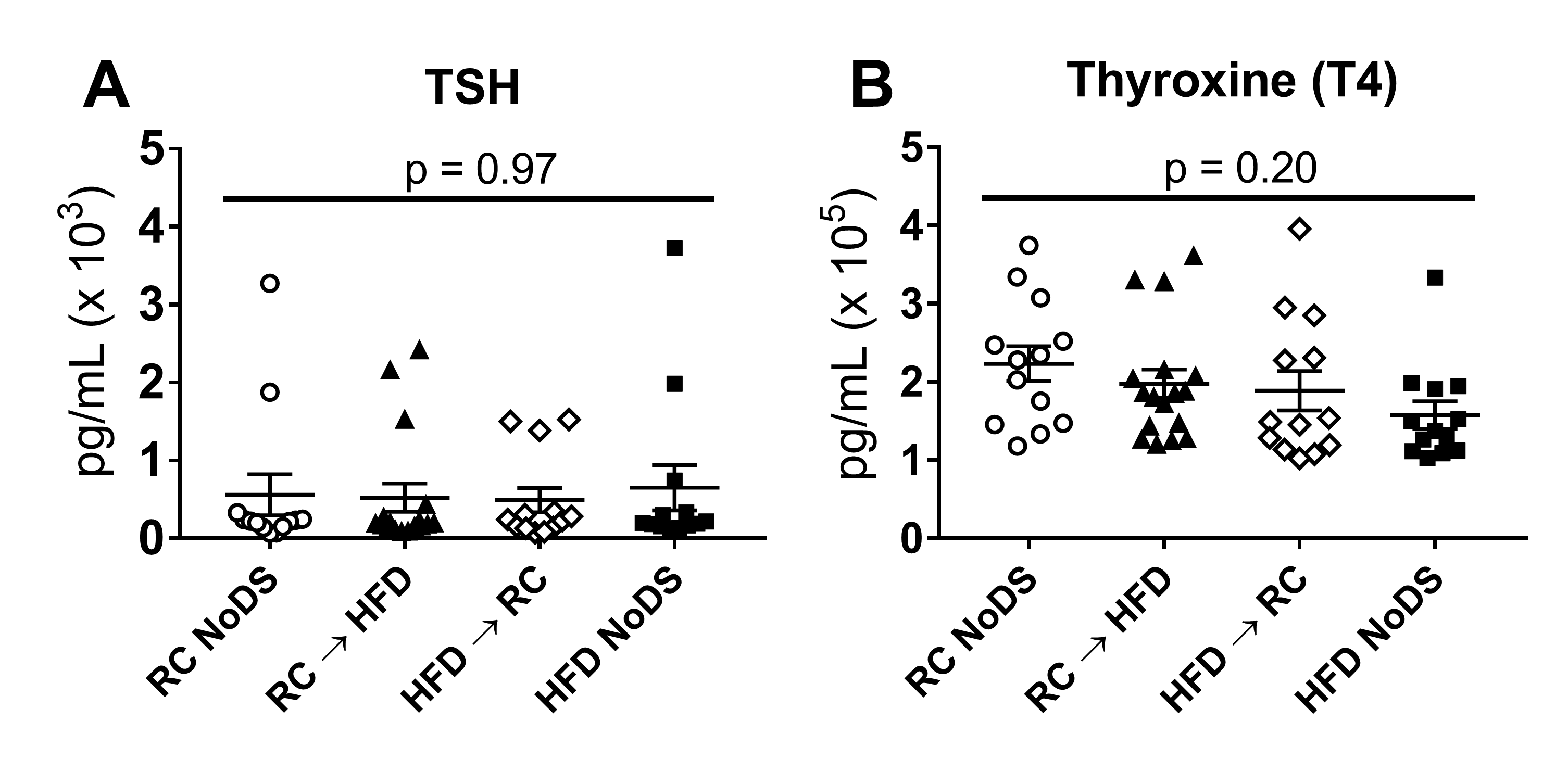

Supplement: S4 Fig — (A) Thyroid-stimulating hormone (TSH) showed highly variable expression levels across samples, contributing to non-significant group effects (p = 0.9658). (B) Thyroxine (T4) levels were not robustly different between dietary conditions. Triiodothyronine (T3) levels were also measured, but most samples were below detection threshold and not quantifiable (not shown). TSH sample sizes: [RC NoDS, n = 11; RC → HFD, n = 14; HFD → RC, n = 11; HFD NoDS, n = 11]. T4 sample sizes: [RC NoDS, n = 13; RC → HFD, n = 17; HFD → RC, n = 13; HFD NoDS, n = 13]. (TIF) [file pone.0196743.s004.tif]

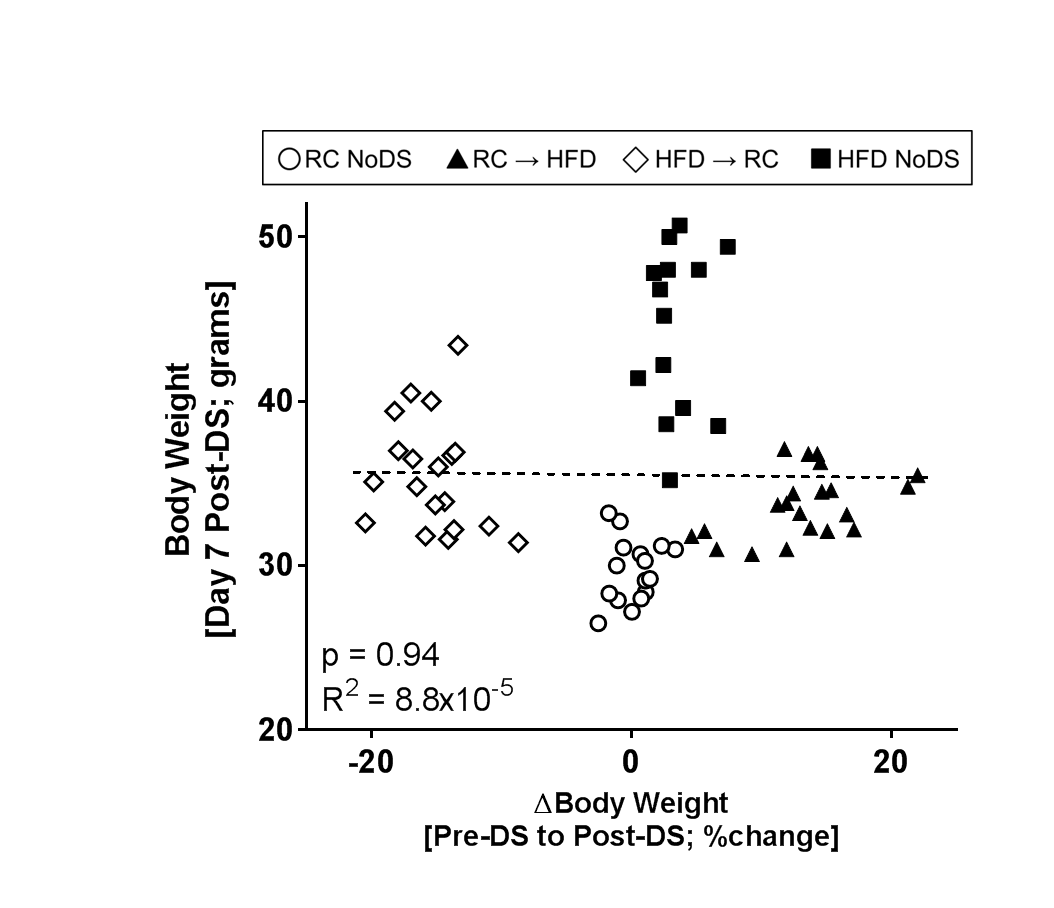

Supplement: S5 Fig — Scatter plot of absolute body weight (Post-DS, in grams) vs Δbody weight (percent change in body weight from Pre- to Post-DS). There is no correlation between body weight and Δbody weight for these mice across all groups (R2 = 8.8x10^-5^, p = 0.94). RC NoDS, n = 16; RC → HFD, n = 21; HFD → RC, n = 19; HFD NoDS, n = 14. (TIF) [file pone.0196743.s005.tif]

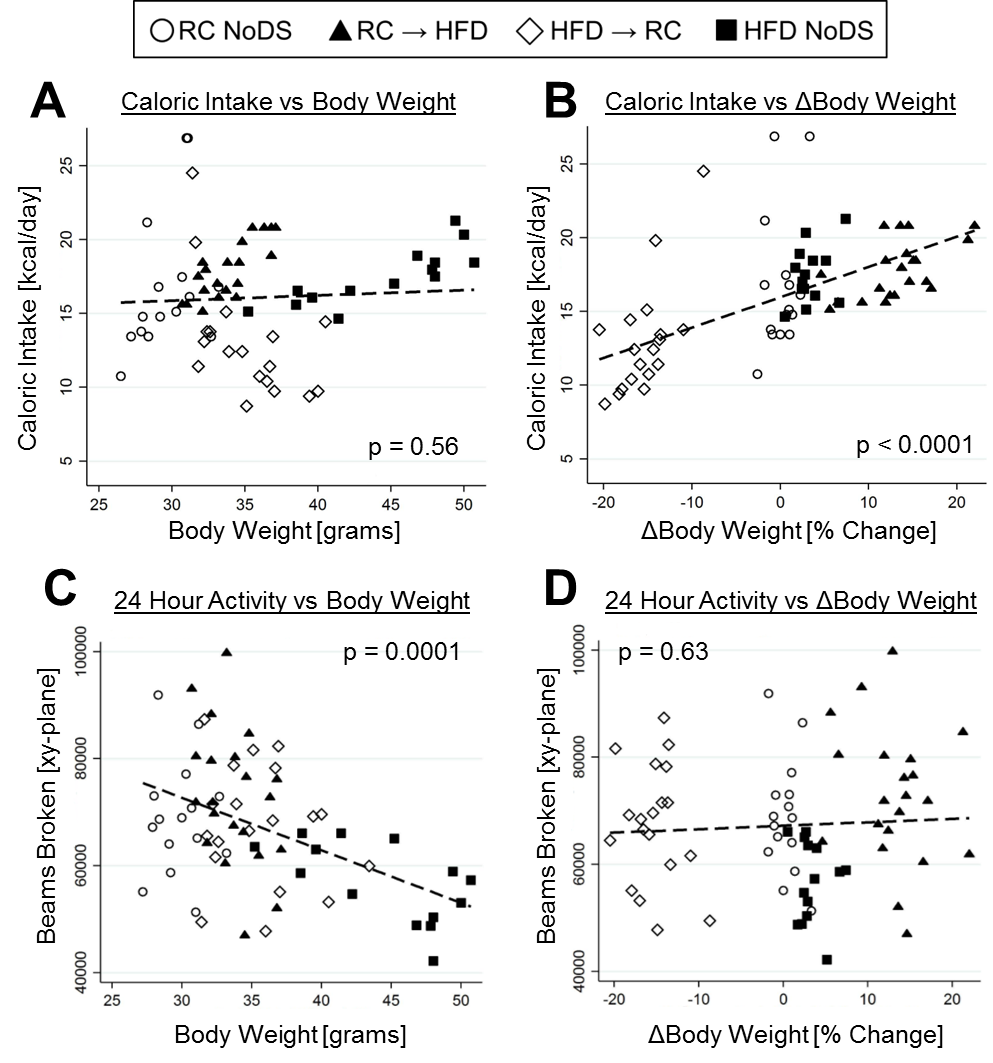

Supplement: S6 Fig — (A) Caloric intake is not related to body weight. (B) Energy intake is positively related to acute weight changes (Δbody weight). (C) Activity patterns are negatively associated with body weight. (D) Locomotor activity is not related to Δbody weight. (TIF) [file pone.0196743.s006.tif]

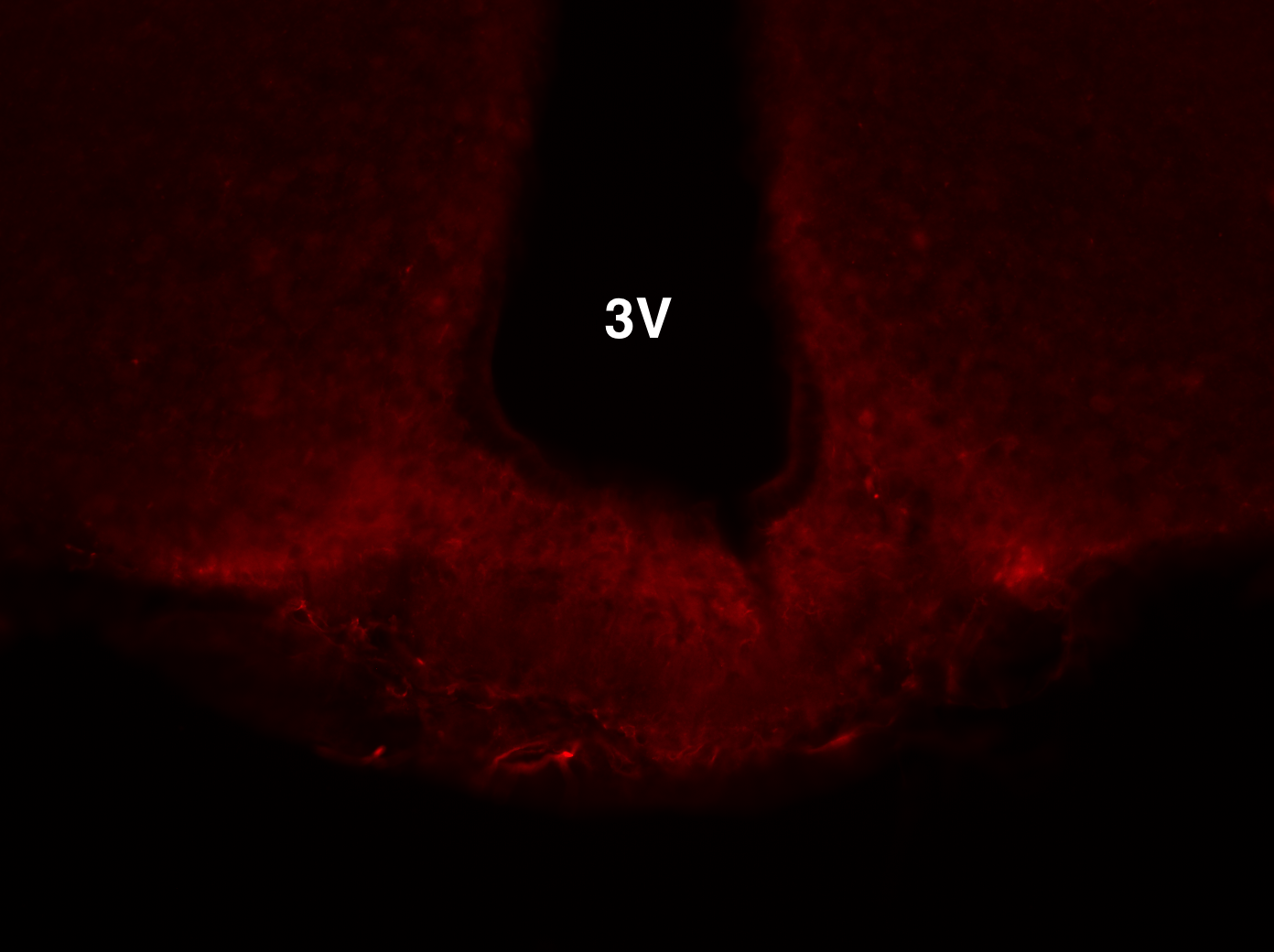

Supplement: S7 Fig — One mouse was deeply anesthetized and transcardially perfused with ice-cold 4% paraformaldehyde (pH = 7.5). The brain was left in 4% PF overnight, and then switched to 30% sucrose the following day. A cryostat was used to coronally section the brain (50 μm) and free-floating sections were placed into blocking buffer (4% normal donkey serum and 0.4% Triton-X in 1x PBS) overnight. The following day, anti-NeuN (1:1000, MAB377, EMD Millipore) was diluted in blocking buffer. Brain slices were incubated in primary antibody at 4°C on a shaker for 3 days. The primary was then washed off thrice with 1X PBS, and the secondary antibodies (A21206, Invitrogen, Carlsbad, CA) was applied (1:500 in 50% blocking buffer:50% 1X PBS) for 1 hour. Image was taken at 20x magnification. (TIF) [file pone.0196743.s007.tif]
