# Supplementary material for: Dietary challenges differentially affect activity and sleep/wake behavior in mus musculus: Isolating independent associations with diet/energy balance and body weight
Source: PLoS One. 2018 May 10;13(5):e0196743. doi: 10.1371/journal.pone.0196743 (PMC5945034; doi:10.1371/journal.pone.0196743)
Supplement: S1 Table — (DOCX) [file pone.0196743.s008.docx]

| **Sample ID** | **Total Reads [#]** | **Ribosomal RNA** | | **Mitochondrial DNA** | |
| --- | --- | --- | --- | --- | --- |
|  |  | **Unique Reads [#]** | **Unique Reads [%]** | **Unique Reads [#]** | **Unique Reads [%]** |
| RC NoDS.01 | 39,976,549 | 914,784 | 2.28 | 719,521 | 1.80 |
| RC NoDS.02 | 35,798,997 | 1,186,847 | 3.31 | 568,321 | 1.60 |
| RC NoDS.03 | 47,083,916 | 1,553,233 | 3.29 | 650,136 | 1.40 |
| RC NoDS.05 | 40,940,308 | 1,230,467 | 3.00 | 390,894 | 0.90 |
| RC NoDS.06 | 45,770,168 | 1,615,528 | 3.52 | 388,202 | 0.80 |
| RC-HFD.07 | 38,781,692 | 906,187 | 2.33 | 547,978 | 1.40 |
| RC-HFD.08 | 42,003,016 | 1,865,562 | 4.44 | 615,024 | 1.50 |
| RC-HFD.09 | 51,745,981 | 1,463,758 | 2.82 | 647,418 | 1.20 |
| RC-HFD.10 | 49,316,975 | 2,876,939 | 5.83 | 606,875 | 1.20 |
| RC-HFD.11 | 58,975,589 | 2,097,050 | 3.55 | 599,556 | 1.00 |
| RC-HFD.12 | 45,443,568 | 2,086,999 | 4.59 | 523,198 | 1.10 |
| HFD-RC.13 | 20,984,913 | 656,285 | 3.12 | 224,815 | 1.10 |
| HFD-RC.14 | 53,072,720 | 1,640,727 | 3.09 | 568,618 | 1.10 |
| HFD-RC.15 | 59,752,128 | 2,292,245 | 3.83 | 760,391 | 1.30 |
| HFD-RC.16 | 61,245,611 | 2,386,657 | 3.89 | 839,802 | 1.40 |
| HFD-RC.17 | 52,892,796 | 1,436,217 | 2.71 | 669,739 | 1.30 |
| HFD NoDS.19 | 55,625,859 | 2,514,734 | 4.52 | 499,525 | 0.90 |
| HFD NoDS.20 | 44,776,843 | 2,706,141 | 6.04 | 219,408 | 0.50 |
| HFD NoDS.21 | 55,935,831 | 3,387,002 | 6.05 | 752,085 | 1.30 |
| HFD NoDS.23 | 48,446,179 | 1,979,114 | 4.08 | 605,161 | 1.20 |
| HFD NoDS.24 | 47,731,405 | 1,289,909 | 2.70 | 583,758 | 1.20 |
| **MINIMUM** | **20,984,913** | **656,285** | **2.28** | **219,408** | **0.50** |
| **MAXIMUM** | **61,245,611** | **3,387,002** | **6.05** | **839,802** | **1.80** |
