# Supplementary material for: Dietary challenges differentially affect activity and sleep/wake behavior in mus musculus: Isolating independent associations with diet/energy balance and body weight
Source: PLoS One. 2018 May 10;13(5):e0196743. doi: 10.1371/journal.pone.0196743 (PMC5945034; doi:10.1371/journal.pone.0196743)
Supplement: S2 Table — Using the ‘High Filter’ data set of RNA-seq results (see Methods), GSEA reveals significantly enriched pathways in HFD → RC animals compared to RC → HFD. Using both raw and log2-transformed data, we found the same six enriched pathways at an FWER < 0.05. FDR: False discovery rate; FWER: Family wise error rate; PPARg: Peroxisome proliferator-activated receptor gamma. (DOCX) [file pone.0196743.s009.docx]

| **GSEA Pathway Name** | **Log_2_-Transformed** | | **Raw values** | | **General Description** |
| --- | --- | --- | --- | --- | --- |
|  | **FDR**  **q-value** | **FWER**  **p-value** | **FDR**  **q-value** | **FWER**  **p-value** |  |
| Oxidative Phosphorylation | <0.0001 | <0.0001 | <0.0001 | <0.0001 | Mitochondrial process to aerobically generate ATP |
| Coagulation | 0.017 | 0.020 | 0.009 | 0.016 | Generally involved in clotting blood following injury, ‘coagulation’ may suggest changes to brain vasculature in circumventricular regions |
| KRAS Signaling Up | 0.021 | 0.048 | 0.015 | 0.05 | KRAS is an oncogene that regulates protein dynamics and glucose homeostasis |
| Adipogenesis | 0.020 | 0.035 | 0.033 | 0.11 | The formation of mature adipocytes, which store fatty acids. This complex process is crucially regulated by PPARg |
| Estrogen Response  Late | 0.017 | 0.050 | 0.015 | 0.044 | Genes which are upregulated by the late response phase following estrogen hormone |
| Xenobiotic Metabolism | 0.026 | 0.092 | 0.006 | 0.006 | Process to break down and dispose of foreign substances, such as drugs or food additives |
