# Supplementary material for: Dietary challenges differentially affect activity and sleep/wake behavior in mus musculus: Isolating independent associations with diet/energy balance and body weight
Source: PLoS One. 2018 May 10;13(5):e0196743. doi: 10.1371/journal.pone.0196743 (PMC5945034; doi:10.1371/journal.pone.0196743)
Supplement: S3 Table — (DOCX) [file pone.0196743.s010.docx]

| **Gene Name** | **NCBI Reference Sequence** | **Catalog Number** |
| --- | --- | --- |
| *Npy* | NM_023456.2 | Mm01410146_m1 |
| *Agrp* | NM_007427.3 | Mm00475829_g1 |
| *Cartpt* | NM_013732.7 | Mm04210469_m1 |
| *Pomc* | NM_008895.4 | Mm00435874_m1 |
| *Trh* | NM_009426.3 | Mm01182425_g1 |
| *Hcrt* | NM_010410.2 | Mm01964030_s1 |
| *Serpina3n* | NM_009252.2 | Mm00776439_m1 |
| *Sbno2* | NM_183426.1 | Mm00839231_m1 |
| *Rn18s* | NR_003278.3 | Mm03928990_g1 |
| *Actb* | NM_007393.5 | Mm00607939_s1 |
| *Rplp0* | NM_007475.5 | Mm00725448_s1 |
