# Supplementary material for: Dietary challenges differentially affect activity and sleep/wake behavior in mus musculus: Isolating independent associations with diet/energy balance and body weight
Source: PLoS One. 2018 May 10;13(5):e0196743. doi: 10.1371/journal.pone.0196743 (PMC5945034; doi:10.1371/journal.pone.0196743)
Supplement: S4 Table — Only comparisons that reached overall significance (ANOVA: p<0.05) are shown. (DOCX) [file pone.0196743.s011.docx]

| **Figure**  **Panel** | **Statistical Test & Main Effect** | | **Post-Hoc Comparison** | **Adjusted**  **p-value** |
| --- | --- | --- | --- | --- |
| **Fig 1** | | | | |
| Fig 1C | One-way ANOVA  F = 55.25  p < 0.0001 | | RC NoDS vs. RC → HFD | 0.0036 |
|  |  |  | RC NoDS vs. HFD → RC | <0.0001 |
|  |  |  | RC NoDS vs. HFD NoDS | <0.0001 |
|  |  |  | RC → HFD vs. HFD → RC | 0.17 |
|  |  |  | RC → HFD vs. HFD NoDS | <0.0001 |
|  |  |  | HFD → RC vs. HFD NoDS | <0.0001 |
|  | | | | |
| Fig 1E | One-way ANOVA  F = 219.8  p < 0.0001 | | RC NoDS vs. RC → HFD | <0.0001 |
|  |  |  | RC NoDS vs. HFD → RC | <0.0001 |
|  |  |  | RC NoDS vs. HFD NoDS | 0.059 |
|  |  |  | RC → HFD vs. HFD → RC | <0.0001 |
|  |  |  | RC → HFD vs. HFD NoDS | <0.0001 |
|  |  |  | HFD → RC vs. HFD NoDS | <0.0001 |
|  | | | | |
| Fig 1G | One-way ANOVA  F = 5.42  p = 0.0021 | | RC NoDS vs. RC → HFD | 0.90 |
|  |  |  | RC NoDS vs. HFD → RC | 0.0033 |
|  |  |  | RC NoDS vs. HFD NoDS | 0.91 |
|  |  |  | RC → HFD vs. HFD → RC | 0.012 |
|  |  |  | RC → HFD vs. HFD NoDS | >0.99 |
|  |  |  | HFD → RC vs. HFD NoDS | 0.036 |
| **Fig 2** | | | | |
| Fig 2C | One-way ANOVA  F = 5.92  p = 0.0012 | | RC NoDS vs. RC → HFD | 0.73 |
|  |  |  | RC NoDS vs. HFD → RC | 0.98 |
|  |  |  | RC NoDS vs. HFD NoDS | 0.025 |
|  |  |  | RC → HFD vs. HFD → RC | 0.45 |
|  |  |  | RC → HFD vs. HFD NoDS | 0.0006 |
|  |  |  | HFD → RC vs. HFD NoDS | 0.041 |
| **Fig 3** | | | | |
| Fig 3A | One-way ANOVA  F = 11.96  p = 0.0002 | | RC NoDS vs. RC → HFD | 0.0002 |
|  |  |  | RC NoDS vs. HFD → RC | 0.34 |
|  |  |  | RC NoDS vs. HFD NoDS | 0.0020 |
|  |  |  | RC → HFD vs. HFD → RC | 0.017 |
|  |  |  | RC → HFD vs. HFD NoDS | 0.83 |
|  |  |  | HFD → RC vs. HFD NoDS | 0.11 |
|  | | | | |
| Fig 3B | One-way ANOVA  F = 9.65  p < 0.0001 | | RC NoDS vs. RC → HFD | <0.0001 |
|  |  |  | RC NoDS vs. HFD → RC | 0.65 |
|  |  |  | RC NoDS vs. HFD NoDS | 0.0074 |
|  |  |  | RC → HFD vs. HFD → RC | 0.0085 |
|  |  |  | RC → HFD vs. HFD NoDS | 0.98 |
|  |  |  | HFD → RC vs. HFD NoDS | 0.11 |
|  | | | | |
| Fig 3C | One-way ANOVA  F = 5.45  p = 0.0076 | | RC NoDS vs. RC → HFD | 0.027 |
|  |  |  | RC NoDS vs. HFD → RC | 0.99 |
|  |  |  | RC NoDS vs. HFD NoDS | 0.084 |
|  |  |  | RC → HFD vs. HFD → RC | 0.035 |
|  |  |  | RC → HFD vs. HFD NoDS | >0.99 |
|  |  |  | HFD → RC vs. HFD NoDS | 0.11 |
|  | | | | |
| Fig 3D | One-way ANOVA  F = 22.3  p < 0.0001 | | RC NoDS vs. RC → HFD | <0.0001 |
|  |  |  | RC NoDS vs. HFD → RC | 0.069 |
|  |  |  | RC NoDS vs. HFD NoDS | <0.0001 |
|  |  |  | RC → HFD vs. HFD → RC | 0.0026 |
|  |  |  | RC → HFD vs. HFD NoDS | >0.99 |
|  |  |  | HFD → RC vs. HFD NoDS | 0.0057 |
|  | | | | |
| Fig 3E | One-way ANOVA  F = 24.4  p < 0.0001 | | RC NoDS vs. RC → HFD | <0.0001 |
|  |  |  | RC NoDS vs. HFD → RC | 0.056 |
|  |  |  | RC NoDS vs. HFD NoDS | <0.0001 |
|  |  |  | RC → HFD vs. HFD → RC | <0.0001 |
|  |  |  | RC → HFD vs. HFD NoDS | 0.60 |
|  |  |  | HFD → RC vs. HFD NoDS | 0.043 |
|  | | | | |
| Fig 3F | One-way ANOVA  F = 11.88  p = 0.0002 | | RC NoDS vs. RC → HFD | 0.0004 |
|  |  |  | RC NoDS vs. HFD → RC | 0.73 |
|  |  |  | RC NoDS vs. HFD NoDS | 0.011 |
|  |  |  | RC → HFD vs. HFD → RC | 0.0024 |
|  |  |  | RC → HFD vs. HFD NoDS | 0.96 |
|  |  |  | HFD → RC vs. HFD NoDS | 0.055 |
|  | | | | |
| Fig 3G | One-way ANOVA  F = 17.12  p < 0.0001 | | RC NoDS vs. RC → HFD | 0.071 |
|  |  |  | RC NoDS vs. HFD → RC | 0.88 |
|  |  |  | RC NoDS vs. HFD NoDS | <0.0001 |
|  |  |  | RC → HFD vs. HFD → RC | 0.27 |
|  |  |  | RC → HFD vs. HFD NoDS | 0.0032 |
|  |  |  | HFD → RC vs. HFD NoDS | 0.0001 |
|  | | | | |
| Fig 3H | One-way ANOVA  F = 5.79  p = 0.0020 | | RC NoDS vs. RC → HFD | 0.47 |
|  |  |  | RC NoDS vs. HFD → RC | 0.59 |
|  |  |  | RC NoDS vs. HFD NoDS | 0.0009 |
|  |  |  | RC → HFD vs. HFD → RC | >0.99 |
|  |  |  | RC → HFD vs. HFD NoDS | 0.015 |
|  |  |  | HFD → RC vs. HFD NoDS | 0.026 |
|  | | | | |
| Fig 3I | One-way ANOVA  F = 4.47  p = 0.016 | | RC NoDS vs. RC → HFD | 0.39 |
|  |  |  | RC NoDS vs. HFD → RC | 0.15 |
|  |  |  | RC NoDS vs. HFD NoDS | 0.011 |
|  |  |  | RC → HFD vs. HFD → RC | 0.86 |
|  |  |  | RC → HFD vs. HFD NoDS | 0.098 |
|  |  |  | HFD → RC vs. HFD NoDS | 0.32 |
|  | | | | |
| Fig 3J | One-way ANOVA  F = 18.66  p < 0.0001 | | RC NoDS vs. RC → HFD | 0.0091 |
|  |  |  | RC NoDS vs. HFD → RC | 0.0025 |
|  |  |  | RC NoDS vs. HFD NoDS | <0.0001 |
|  |  |  | RC → HFD vs. HFD → RC | 0.85 |
|  |  |  | RC → HFD vs. HFD NoDS | 0.0039 |
|  |  |  | HFD → RC vs. HFD NoDS | 0.027 |
|  | | | | |
| Fig 3K | One-way ANOVA  F = 7.14  p = 0.0005 | | RC NoDS vs. RC → HFD | 0.040 |
|  |  |  | RC NoDS vs. HFD → RC | 0.0056 |
|  |  |  | RC NoDS vs. HFD NoDS | 0.0008 |
|  |  |  | RC → HFD vs. HFD → RC | 0.68 |
|  |  |  | RC → HFD vs. HFD NoDS | 0.16 |
|  |  |  | HFD → RC vs. HFD NoDS | 0.71 |
|  | | | | |
| Fig 3L | One-way ANOVA  F = 3.28  p = 0.045 | | RC NoDS vs. RC → HFD | >0.99 |
|  |  |  | RC NoDS vs. HFD → RC | 0.35 |
|  |  |  | RC NoDS vs. HFD NoDS | 0.084 |
|  |  |  | RC → HFD vs. HFD → RC | 0.33 |
|  |  |  | RC → HFD vs. HFD NoDS | 0.072 |
|  |  |  | HFD → RC vs. HFD NoDS | 0.64 |
| **Fig 4** | | | | |
| Fig 4A | One-way ANOVA  F = 26.24  p < 0.0001 | | RC NoDS vs. RC → HFD | <0.0001 |
|  |  |  | RC NoDS vs. HFD → RC | 0.96 |
|  |  |  | RC NoDS vs. HFD NoDS | <0.0001 |
|  |  |  | RC → HFD vs. HFD → RC | <0.0001 |
|  |  |  | RC → HFD vs. HFD NoDS | 0.61 |
|  |  |  | HFD → RC vs. HFD NoDS | <0.0001 |
|  | | | | |
| Fig 4B | One-way ANOVA  F = 9.38  p < 0.0001 | | RC NoDS vs. RC → HFD | 0.10 |
|  |  |  | RC NoDS vs. HFD → RC | 0.98 |
|  |  |  | RC NoDS vs. HFD NoDS | <0.0001 |
|  |  |  | RC → HFD vs. HFD → RC | 0.25 |
|  |  |  | RC → HFD vs. HFD NoDS | 0.041 |
|  |  |  | HFD → RC vs. HFD NoDS | 0.0004 |
|  | | | | |
| Fig 4C | One-way ANOVA  F = 18.99  p < 0.0001 | | RC NoDS vs. RC → HFD | 0.0016 |
|  |  |  | RC NoDS vs. HFD → RC | 0.028 |
|  |  |  | RC NoDS vs. HFD NoDS | <0.0001 |
|  |  |  | RC → HFD vs. HFD → RC | 0.85 |
|  |  |  | RC → HFD vs. HFD NoDS | 0.0009 |
|  |  |  | HFD → RC vs. HFD NoDS | 0.0002 |
| **S3 Fig** | | | | |
| Fig S3A | | One-way ANOVA  F = 15.93  p < 0.0001 | RC NoDS vs. RC → HFD | 0.45 |
|  |  |  | RC NoDS vs. HFD → RC | 0.95 |
|  |  |  | RC NoDS vs. HFD NoDS | <0.0001 |
|  |  |  | RC → HFD vs. HFD → RC | 0.77 |
|  |  |  | RC → HFD vs. HFD NoDS | 0.0007 |
|  |  |  | HFD → RC vs. HFD NoDS | 0.0002 |
|  | | | | |
| Fig S3B | | One-way ANOVA  F = 4.60  p = 0.0069 | RC NoDS vs. RC → HFD | 0.85 |
|  |  |  | RC NoDS vs. HFD → RC | 0.22 |
|  |  |  | RC NoDS vs. HFD NoDS | 0.0064 |
|  |  |  | RC → HFD vs. HFD → RC | 0.55 |
|  |  |  | RC → HFD vs. HFD NoDS | 0.023 |
|  |  |  | HFD → RC vs. HFD NoDS | 0.34 |
|  | | | | |
| Fig S3D | | One-way ANOVA  F = 12.17  p = 0.0002 | RC NoDS vs. RC → HFD | 0.46 |
|  |  |  | RC NoDS vs. HFD → RC | 0.69 |
|  |  |  | RC NoDS vs. HFD NoDS | 0.0002 |
|  |  |  | RC → HFD vs. HFD → RC | 0.98 |
|  |  |  | RC → HFD vs. HFD NoDS | 0.0022 |
|  |  |  | HFD → RC vs. HFD NoDS | 0.0016 |
|  | | | | |
| Fig S3E | | One-way ANOVA  F = 7.16  p = 0.0005 | RC NoDS vs. RC → HFD | 0.80 |
|  |  |  | RC NoDS vs. HFD → RC | 0.43 |
|  |  |  | RC NoDS vs. HFD NoDS | 0.0003 |
|  |  |  | RC → HFD vs. HFD → RC | 0.87 |
|  |  |  | RC → HFD vs. HFD NoDS | 0.0015 |
|  |  |  | HFD → RC vs. HFD NoDS | 0.020 |
| **S4 Fig** | | | | |
| Fig S4A | | One-way ANOVA  F = 11.97  p = 0.0002 | RC NoDS vs. RC → HFD | 0.23 |
|  |  |  | RC NoDS vs. HFD → RC | 0.031 |
|  |  |  | RC NoDS vs. HFD NoDS | 0.18 |
|  |  |  | RC → HFD vs. HFD → RC | 0.0004 |
|  |  |  | RC → HFD vs. HFD NoDS | >0.99 |
|  |  |  | HFD → RC vs. HFD NoDS | 0.0004 |
|  | | | | |
| Fig S4B | | One-way ANOVA  F = 12.1  p = 0.0002 | RC NoDS vs. RC → HFD | 0.0045 |
|  |  |  | RC NoDS vs. HFD → RC | 0.36 |
|  |  |  | RC NoDS vs. HFD NoDS | 0.63 |
|  |  |  | RC → HFD vs. HFD → RC | 0.0001 |
|  |  |  | RC → HFD vs. HFD NoDS | 0.059 |
|  |  |  | HFD → RC vs. HFD NoDS | 0.45 |
|  | | | | |
| Fig S4C | | One-way ANOVA  F = 8.03  p = 0.0015 | RC NoDS vs. RC → HFD | 0.44 |
|  |  |  | RC NoDS vs. HFD → RC | 0.026 |
|  |  |  | RC NoDS vs. HFD NoDS | 0.96 |
|  |  |  | RC → HFD vs. HFD → RC | 0.0008 |
|  |  |  | RC → HFD vs. HFD NoDS | 0.22 |
|  |  |  | HFD → RC vs. HFD NoDS | 0.065 |
|  | | | | |
| Fig S4D | | One-way ANOVA  F = 22.78  p < 0.0001 | RC NoDS vs. RC → HFD | 0.70 |
|  |  |  | RC NoDS vs. HFD → RC | 0.13 |
|  |  |  | RC NoDS vs. HFD NoDS | 0.0001 |
|  |  |  | RC → HFD vs. HFD → RC | 0.012 |
|  |  |  | RC → HFD vs. HFD NoDS | 0.0008 |
|  |  |  | HFD → RC vs. HFD NoDS | <0.0001 |
|  | | | | |
| Fig S4E | | One-way ANOVA  F = 15.69  p < 0.0001 | RC NoDS vs. RC → HFD | <0.0001 |
|  |  |  | RC NoDS vs. HFD → RC | 0.070 |
|  |  |  | RC NoDS vs. HFD NoDS | 0.0085 |
|  |  |  | RC → HFD vs. HFD → RC | 0.0049 |
|  |  |  | RC → HFD vs. HFD NoDS | 0.046 |
|  |  |  | HFD → RC vs. HFD NoDS | 0.72 |
|  | | | | |
| Fig S4F | | One-way ANOVA  F = 9.00  p = 0.0009 | RC NoDS vs. RC → HFD | >0.99 |
|  |  |  | RC NoDS vs. HFD → RC | 0.23 |
|  |  |  | RC NoDS vs. HFD NoDS | 0.0021 |
|  |  |  | RC → HFD vs. HFD → RC | 0.20 |
|  |  |  | RC → HFD vs. HFD NoDS | 0.0014 |
|  |  |  | HFD → RC vs. HFD NoDS | 0.12 |
